# Effective Respiratory CD8 T-Cell Immunity to Influenza Virus Induced by Intranasal Carbomer-Lecithin-Adjuvanted Non-replicating Vaccines

**DOI:** 10.1371/journal.ppat.1006064

**Published:** 2016-12-20

**Authors:** David J. Gasper, Brandon Neldner, Erin H. Plisch, Hani Rustom, Emily Carrow, Hirotaka Imai, Yoshihiro Kawaoka, M. Suresh

**Affiliations:** 1 Department of Pathobiological Sciences, School of Veterinary Medicine, University of Wisconsin-Madison, Madison, Wisconsin, United States of America; 2 Comparative Biomedical Sciences Graduate Program, School of Veterinary Medicine, University of Wisconsin-Madison, Madison, Wisconsin, United States of America; 3 Advanced Bioadjuvants, Omaha, Nebraska, United States of America; Nationwide Children's Hospital, UNITED STATES

## Abstract

CD8^+^ cytotoxic T lymphocytes (CTLs) are critical for clearing many viral infections, and protective CTL memory can be induced by vaccination with attenuated viruses and vectors. Non-replicating vaccines are typically potentiated by the addition of adjuvants that enhance humoral responses, however few are capable of generating CTL responses. Adjuplex is a carbomer-lecithin-based adjuvant demonstrated to elicit robust humoral immunity to non-replicating antigens. We report that mice immunized with non-replicating Adjuplex-adjuvanted vaccines generated robust antigen-specific CTL responses. Vaccination by the subcutaneous or the intranasal route stimulated systemic and mucosal CTL memory respectively. However, only CTL memory induced by intranasal vaccination was protective against influenza viral challenge, and correlated with an enhancement of memory CTLs in the airways and CD103^+^ CD69^+^ CXCR3^+^ resident memory-like CTLs in the lungs. Mechanistically, Myd88-deficient mice mounted primary CTL responses to Adjuplex vaccines that were similar in magnitude to wild-type mice, but exhibited altered differentiation of effector cell subsets. Immune potentiating effects of Adjuplex entailed alterations in the frequency of antigen-presenting-cell subsets in vaccine draining lymph nodes, and in the lungs and airways following intranasal vaccination. Further, Adjuplex enhanced the ability of dendritic cells to promote antigen-induced proliferation of naïve CD8 T cells by modulating antigen uptake, its intracellular localization, and rate of processing. Taken together, we have identified an adjuvant that elicits both systemic and mucosal CTL memory to non-replicating antigens, and engenders protective CTL-based heterosubtypic immunity to influenza A virus in the respiratory tract. Further, findings presented in this manuscript have provided key insights into the mechanisms and factors that govern the induction and programming of systemic and protective memory CTLs in the respiratory tract.

## Author Summary

Current respiratory-virus vaccines typically employ non-replicating antigens and rely solely on the generation of humoral responses for protection. Viruses such as influenza can mutate and escape these responses, thereby limiting immunity and necessitating revaccination. Cell-mediated immunity (CMI) could provide broader protection by targeting viral components that infrequently mutate, however non-replicating vaccines capable of inducing CMI are not available. Impediments to vaccine development include an incomplete understanding of the nature of protective respiratory CMI and a lack of vaccine adjuvants capable of eliciting CMI to non-replicating antigens. Using a mouse model, we characterized the protective immunity afforded by CMI responses to non-replicating vaccines formulated with the adjuvant Adjuplex. We found that vaccination via either the subcutaneous or intranasal route was capable of inducing potent CMI responses. However, only intranasal vaccination protected against challenge with heterosubtypic influenza viruses. This protection correlated with enhancement of T cells with a resident-memory phenotype in the lungs. Additionally, mechanistic studies showed that Adjuplex affects antigen-presenting cells via activation and alteration of antigen uptake, processing, and presentation. The current studies: (1) identified an adjuvant that elicits protective CMI to respiratory viral pathogens; (2) suggested that stimulation of protective CMI in the respiratory tract requires intranasal vaccine delivery.

## Introduction

Vaccination is the most effective tool for protecting humans and animals from infectious diseases.[1–4] However, despite decades of research, there are no broadly protective vaccines against seasonal influenza A viruses (IAV), and effective vaccines against most other respiratory viruses do not exist. The most effective IAV vaccines currently licensed in the U.S. depend upon the generation of neutralizing antibodies targeting IAV hemagglutinin (HA) antigens.[5] These neutralizing antibodies are capable of eliciting varying levels of protective immunity to specific viruses in certain demographics. However, HA is also the most frequently mutated of the IAV proteins, and the immunity resulting from this year’s vaccine strain may not confer immunity against strains emerging during the current and subsequent influenza seasons. Therefore, vaccine strains must be adjusted annually to match HA predicted for the next influenza season. Even with annual administration, humoral immune responses tend to be short-lived, cross-protection against strains with minor HA mutations is highly variable, and there is progressively less protection against heterosubtypic or heterotypic viruses.[5–8] As a result, current public health policy is largely dependent on annual re-vaccination for seasonal IAV, and pandemic disease surveillance, outbreak containment, and the activation of an emergency vaccine development pipeline aimed at producing a vaccine bespoke for the virus of interest.[9, 10]

IAV vaccines that elicit cell-mediated immunity (CMI) or balanced CMI and antibody responses are promising alternatives to antibody-only strategies.[5, 11–18] Because of their capacity to selectively target and kill IAV-infected cells, CD8^+^ cytotoxic T lymphocytes (CTLs) play a crucial role in the initial clearance of influenza virus infections and are the primary target for most CMI vaccination strategies.[14, 15, 19] [20] Unlike most neutralizing antibodies, CTLs intrinsically target a variety of IAV structural epitopes such as nucleoprotein peptides that are substantially less mutable and more broadly conserved than HA, and they can generate long-lived memory cells capable of mounting cross-protective recall responses.[18, 21–23] Important experimental studies of cell-mediated immunity in mice demonstrate that, separately, influenza-specific memory CTLs and T_H_ cells are sufficient to protect against heterosubtypic influenza challenge.[24] Additionally, naturally occurring cross-protective memory CTL responses in humans are potent enough to be a confounding factor in the evaluation of human IAV challenge studies, and there is significant evidence that pre-existing cross-protective cell-mediated immunity mitigated the effects of the most recent pandemic H1N1 influenza outbreak.[25] These findings strongly suggest that CTL-mediated immunity may provide the means for universal vaccinations for IAV and other respiratory viruses.

Currently licensed CTL-generating vaccines require the presence of a replicating antigen such as an attenuated virus or vector. However, replicating antigens are contraindicated in several key target groups and have the potential for causing clinically significant disease by several mechanisms.[26, 27] By contrast, inactivated viruses and their subunits are comparatively safe, and can be used in at-risk populations. However, non-replicating antigens are intrinsically poor immunogens that primarily generate humoral responses, even when adjuvants are added to enhance immunogenicity.[26, 28] Few modern adjuvants have been reported to safely elicit cell-mediated immunity, and none of those are currently licensed for routine use in the U.S. [8, 15, 26, 28–38] Most of these adjuvants function as simple ligands for pattern recognition receptors, or are immune stimulating complexes that act on a variety of receptors on many cell types.

Adjuplex (ADJ) is a carbomer-lecithin-based adjuvant demonstrated to elicit robust humoral immunity and T-cell responses to subcutaneous IAV subunit vaccines in mice.[39] Here we report that intramuscular, subcutaneous, and intranasal administration of ADJ-adjuvanted non-replicating antigens generated robust antigen-specific CTL responses in mice. Systemic CTL memory was induced regardless of whether mice were vaccinated via subcutaneous, intramuscular or intranasal routes, however, only intranasal vaccination provided protection against influenza viral challenge, and correlated with an enhancement of CD103^+^ CD69^+^ CXCR3^+^ T_RM_ and airway memory T cells in the lung. Thus, ADJ can be used to induce systemic and/or mucosal CTL memory to non-replicating antigens, and by tailoring the route of vaccine delivery we can engender CTL-based protective immunity against systemic and mucosal pathogens.

## Results

### Adjuplex induces robust expansion of antigen-specific CD8 and CD4 T cells

**Fig 1:**
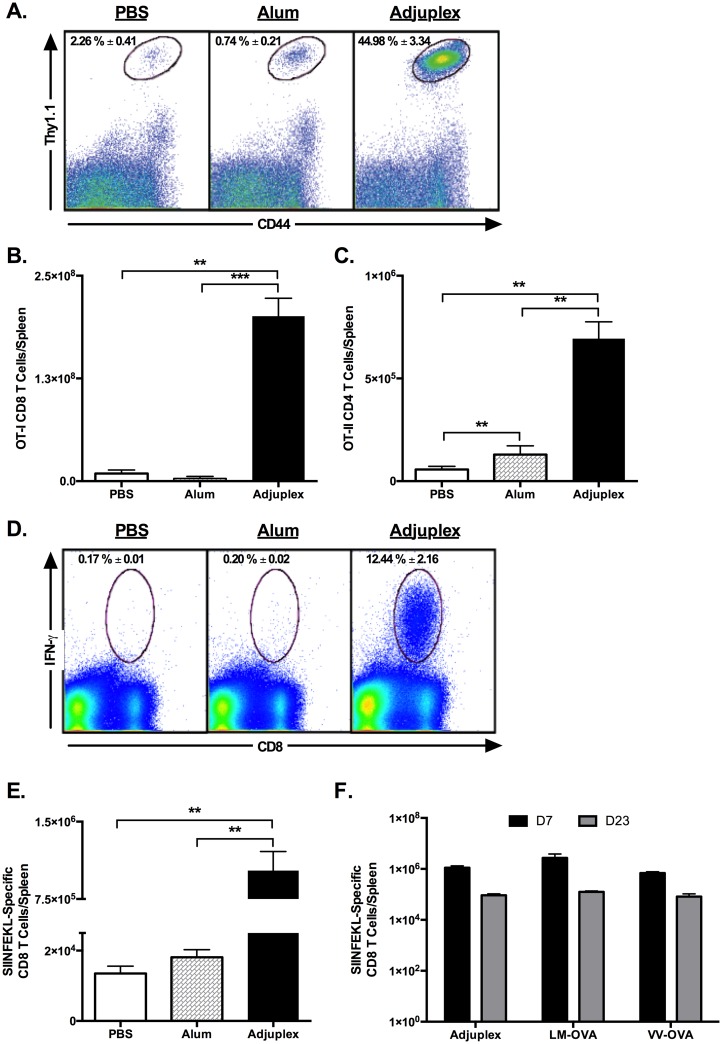
Adjuplex Potently Activates OVA-specific CD8^+^ T cells Following Intramuscular Vaccination. (**A, B, C**) Naïve transgenic OT-I Thy1.1^+^ or OT-II Ly5.1^+^ CD4 T cells were adoptively transferred into Thy1.2^+^ (wild-type) C57BL/6 mice. One day after transfer, the recipient mice were vaccinated by intramuscular injection of 100 μg ovalbumin in PBS alone or supplemented with 50% Imject^™^ Alum, or 20% Adjuplex, and mice were sacrificed after 7 days. Splenocytes were stained with anti-Thy1.1, anti-Ly5.1, anti-CD8, anti-CD4 and anti-CD44. The percentages of CD44^HI^ OT-I or OT-II T cells were quantified by flow cytometry. Dot plots in (**A**) are gated from all splenocytes, and the elliptical gate of interest highlights the frequency of activated (CD44^HI^) OT-I donor cells in the spleen 7 days after vaccination. (**B)** Graph shows the total number of activated OT-I cells in spleen. (**C**) Total numbers of activated OT-II CD4 T cells in spleen. (**D, E)** C57BL/6 mice were vaccinated by intramuscular injection of OVA mixed with PBS, Alum or Adjuplex as above. On day 7 after injection, the numbers of IFN-γ-producing OVA SIINFEKL-specific CD8 T cells in spleen were quantified by intracellular cytokine staining. Plots in (**D**) are gated on viable splenocytes and the numbers are the percentages of IFN-γ-producing cells among CD8 T cells. (**E)** shows the total number of IFN-γ-producing SIINFEKL-specific CD8 T cells in spleen. (**F**) C57BL/6 mice were injected with OVA in Adjuplex or infected with OVA-expressing recombinant *Listeria monocytogenes* (LM-OVA) or vaccinia virus (VV-OVA). At day 7 and 23 post immunization, the numbers of IFN-γ-producing OVA SIINFEKL-specific CD8 T cells in spleen were quantified by intracellular cytokine staining. Figure in **(F)** shows the total number of IFN-γ-producing SIINFEKL-specific CD8 T cells in spleen. Data are from 3–5 mice/group. ** indicates p<0.01, *** indicates p<0.001. Data are representative of two independent experiments.

The carbomer-lecithin based adjuvant ADJ was previously demonstrated to induce humoral responses in a wide range of species.[39–44]. Here, using the tractable chicken ovalbumin (OVA) antigen model, we investigated whether ADJ (confirmed to be endotoxin-free) can elicit antigen-specific CD4 and CD8 T cell responses. OVA-specific naïve CTLs and helper T (T_H_) cells are infrequent in naïve mice, and to enhance assay sensitivity we initially employed adoptive transfer techniques. To assess the activation and expansion of antigen-specific CD8 T cells, we adoptively transferred OVA SIINFEKL-specific naïve TCR transgenic OT-1 CD8 T cells (Thy1.1) into congenic Thy1.2 B6 mice. One day after cell transfer, mice were immunized by intramuscular (I/M) injections with 100 μg OVA in PBS with and without ADJ 20% v/v or Alum 50% v/v. On day 7 after immunization, activated OT-I cells in spleen were enumerated by flow cytometry. Data in Fig 1A and 1B illustrate the potent activation and clonal expansion of donor OT-1 CD8 T cells in spleen of ADJ mice (35–100 fold higher), as compared to OT-I CD8 T cells in mice vaccinated with PBS or Alum.

To assess the activation of OVA-specific CD4 T cells, Ly5.1^+ve^ naïve monoclonal I-A^b^-restricted OVA 323-339-specific TCR Tg OT-II CD4 T cells were adoptively transferred into congenic Ly5.2/B6 mice.^59^ One day after cell transfer, mice were immunized by I/M injections with OVA in PBS with and without ADJ or Alum by I/M injection, as above. On day 7 after immunization, activated OT-II cells in spleen and DLNs were enumerated by flow cytometry. The total number of donor CD44^HI^ OT-II CD4 T cells in spleen of ADJ mice were 5-12-fold greater than in PBS or Alum groups (Fig 1C).

To examine whether ADJ stimulates expansion of polyclonal OVA-specific CD8 T cells, B6 mice were vaccinated by IM injection with OVA with and without adjuvants, as described above. On day 7 after immunization, the numbers of IFNγ-producing OVA epitope SIINFEKL-specific CD8 T cells in spleen were quantified by intracellular cytokine staining (ICCS). As shown in Fig 1D, IFNγ-producing SIINFEKL-specific CD8 T cells were barely detected in spleens of mice from PBS or Alum groups. By contrast, SIINFEKL-specific cytokine-producing CD8 T cells constituted approximately 12% of the CD8 T cells in spleens of mice from the ADJ group. Additionally, the total number of SIINFEKL-specific CD8 T cells in spleen of ADJ-OVA-immunized mice was markedly greater (50 fold) than in the spleen of PBS-OVA- or Alum-OVA-immunized mice (Fig 1E).

We next investigated the degree to which CTL responses to ADJ-OVA vaccination compared to CTL responses elicited by replicating pathogens. Recombinant *Listeria monocytogenes* (LM-OVA) and vaccinia virus (VV-OVA) expressing the OVA have been demonstrated to stimulate strong polyclonal CD8 T cell responses to OVA epitopes.[45–49] We compared the immunogenicity of ADJ-OVA with LM-OVA and VV-OVA. On days 7 and 23 after immunization, the number of SIINFEKL-specific IFNγ-producing CD8 T cells in spleen of ADJ-immunized group was similar to those in LM/OVA and VV/OVA-immunized mice (Fig 1F). Collectively, these data clearly demonstrate that akin to live vaccines, vaccination with ADJ elicited potent monoclonal and polyclonal cell-mediated immune responses to the model antigen OVA.[45–49]

### Magnitude of CD8 T-cell activation correlates with injection site inflammation but is independent of the route of inoculation

**Fig 2:**
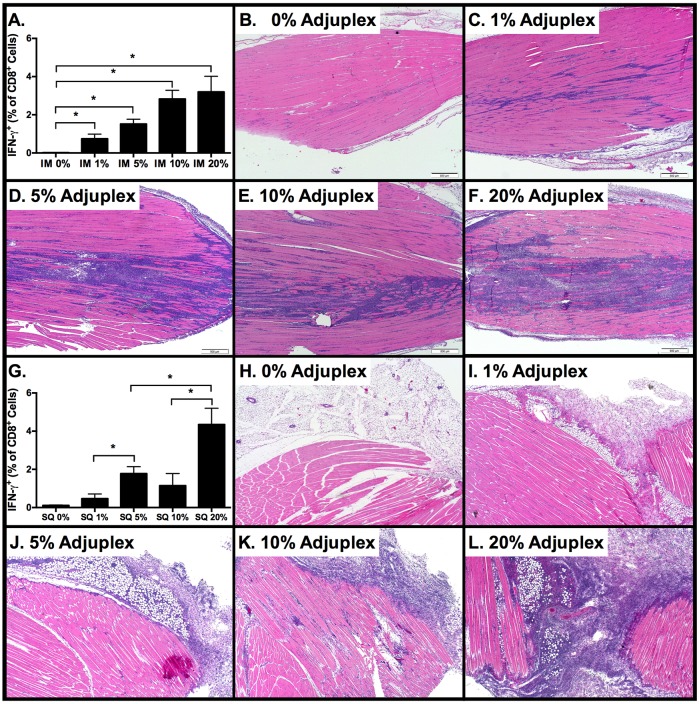
Primary Antigen-Specific CTL Responses Correlate with Adjuplex Dose and Tissue Inflammation. C57BL/6 mice were vaccinated by intramuscular or subcutaneous injection of 100 μg ovalbumin in PBS alone or supplemented with 1, 5, 10 or 20% ADJ. 3–4 mice/group were sacrificed after 7 days. Injection sites were collected for routine histopathology, and spleens were collected for flow cytometry. Prior to FACS analysis, splenocytes were stimulated for 5 hours with SIINFEKL peptide in the presence of IL-2 and brefeldin-A. (**A)** Frequency of SIINFEKL-specific IFN-γ^+^ CD8 T cells among all splenocytes after IM vaccination**. (B-F)** Photomicrographs of histologic changes in the skeletal muscle at the injection site 7 days after IM injection at the indicated concentrations of Adjuplex. Images depict a progressive increase in the cellularity and extent of the inflammatory infiltrate at the injection site. (**G)** Frequency of SIINFEKL-specific IFN-γ^+^ CD8 T cells among all splenocytes after SQ vaccination**.** (**H-L)** Photomicrographs of histologic changes in the subcutaneous adipose tissue and skeletal muscle at the injection site 7 days after SQ injection at the indicated concentrations of Adjuplex. Images depict a progressive increase in the cellularity and extent of the inflammatory infiltrate at the injection site. Data represents two independent experiments.

Inflammation plays a key role in regulating the differentiation of effector and memory CD8 T cells[50]. Therefore, we next sought to determine the effect of ADJ concentration on inflammation and CTL responses to IM vaccination with OVA, and then compare these to CTL responses elicited by subcutaneous (SQ) injection. Mice were immunized IM or SQ with 10 μg OVA in PBS or with ADJ at concentrations of 1, 5, 10, and 20% v/v, and spleen and tissues from injections sites were collected 8 days later. Data in Fig 2A illustrates a near-linear positive correlation between increasing concentrations of IM ADJ and increasing frequency of INF-γ-producing SIINFEKL-specific CTLs in the spleen. Following IM injection with 20% ADJ, frequencies of IFN-γ+ CTLs peaked at 3.2%, however differences between groups that received greater than 1% ADJ were not statistically significant (p>0.05). As demonstrated in the histological images of the injection sites, there was a significant correlation between increasing dose of ADJ and the extent and cellularity of the inflammatory infiltrate at the injection site. The infiltrate was predominantly composed of vacuolated to foamy histiocytes with fewer neutrophils and small lymphocytes, and small foci of coagulative myofiber necrosis were present at the highest doses of ADJ (Fig 2B–2F).

As shown in Fig 2G, the correlation between increasing ADJ concentration and the magnitude of CTL responses to SQ vaccination was similar to IM vaccination. Although the difference between responses to 5% and 10% ADJ concentration was not significant, the differences between responses to 1% vs. 5%, 5% vs. 20%, and 10% vs. 20% were significant (p<0.05). Following SQ vaccination, the ADJ-dose-related increase in the frequency of OVA-specific CTLs again correlated with an increase in the extent and cellularity of the inflammatory infiltrate at the injections, although to a lesser degree than that resulting from IM vaccination. At the highest SQ doses of ADJ, the inflammation extended from the subcutaneous adipose tissue into adjacent muscle (Fig 2H–2L). Based on our results, no more than 5% ADJ is recommended for parenteral use in mice. Taken together, regardless of the route of immunization, 5 or 10% ADJ elicited strong CD8 T cell activation with moderate to low injection site inflammation.

**Fig 3:**
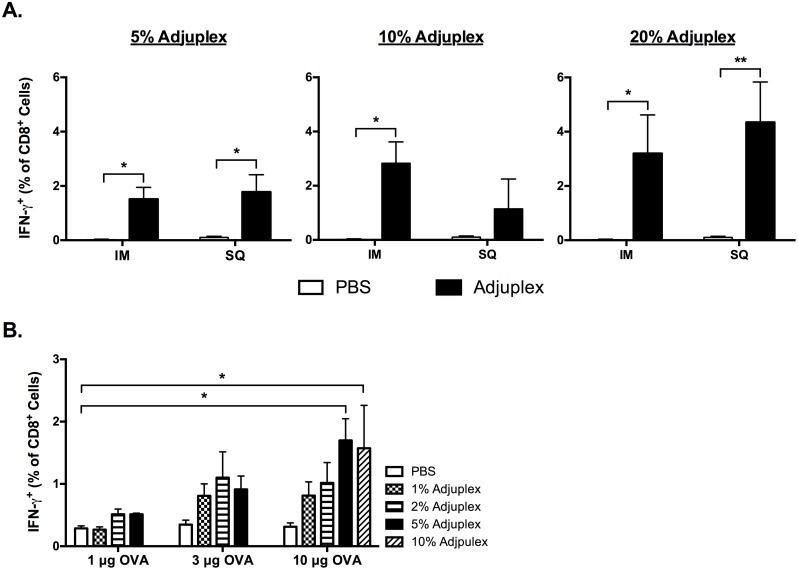
Effect of Vaccination Route and the Dose of Adjuplex and OVA on the Magnitude of CD8 T-cell Responses. (**A**) Intramuscular and subcutaneous vaccination with increasing doses of Adjuplex generate comparable primary CTL responses. WT C57BL/6 mice were vaccinated by intramuscular or subcutaneous injection of 10 μg ovalbumin in PBS alone or mixed with 5, 10 or 20% Adjuplex. At day 7 after vaccination, percentages of SIINFEKL-specific IFN-γ^+^ CD8 T cells in spleen were determined by intracellular staining. (**B**) Primary OVA-specific CTL responses in the spleen are proportionate to SQ vaccination with increasing doses of Adjuplex and OVA. C57BL/6 mice were vaccinated by subcutaneous injection of 1, 3, or 10 μg ovalbumin in PBS alone or supplemented with 1,3, 5 or 10% Adjuplex. The bar graph represents the frequency of SIINFEKL-specific IFN-γ^+^ CD8 T cells among all CD8^+^ splenocytes after vaccination at each dose**.** Data are from analysis of 3–4 mice/group and are representative of two independent experiments.

A pair-wise comparison of IM versus SQ vaccine responses to 10ug OVA at concentrations of 5, 10, and 20% ADJ revealed that the route of vaccination had little effect on the magnitude of the splenic primary CTL responses (Fig 3A). Using the SQ route, we then performed a matrix titration with increasing doses of ADJ with 1, 3, or 10 μg OVA (Fig 3B). Statistically significant increases (p<0.05) in the frequency of IFN-γ-producing SIINFEKL-specific CTLs in the spleen were observed between 1 μg OVA + 0% ADJ, 10 μg OVA + 5% ADJ, and 10 μg OVA + 10% ADJ. There were no significant differences (p>0.05) between any doses lower than 5% ADJ+10 μg OVA when compared to baseline (PBS group, 1 μg OVA). Therefore, we considered responses to only 2 vaccine combinations, 10 ug OVA + 5% ADJ and 10 ug OVA + 10% ADJ, to be above the threshold for reliable detection of an adjuvant effect on systemic CTL responses to SQ vaccination in this system. As there were no significant differences between the magnitude of primary CTL responses to 10 μg OVA at 5 and 10% ADJ, we elected to use 5% ADJ +10 μg OVA for subsequent SQ vaccine studies.

### Primary and memory CTL formation following prime-only vaccination, and CTL-based protection against influenza challenge

**Fig 4:**
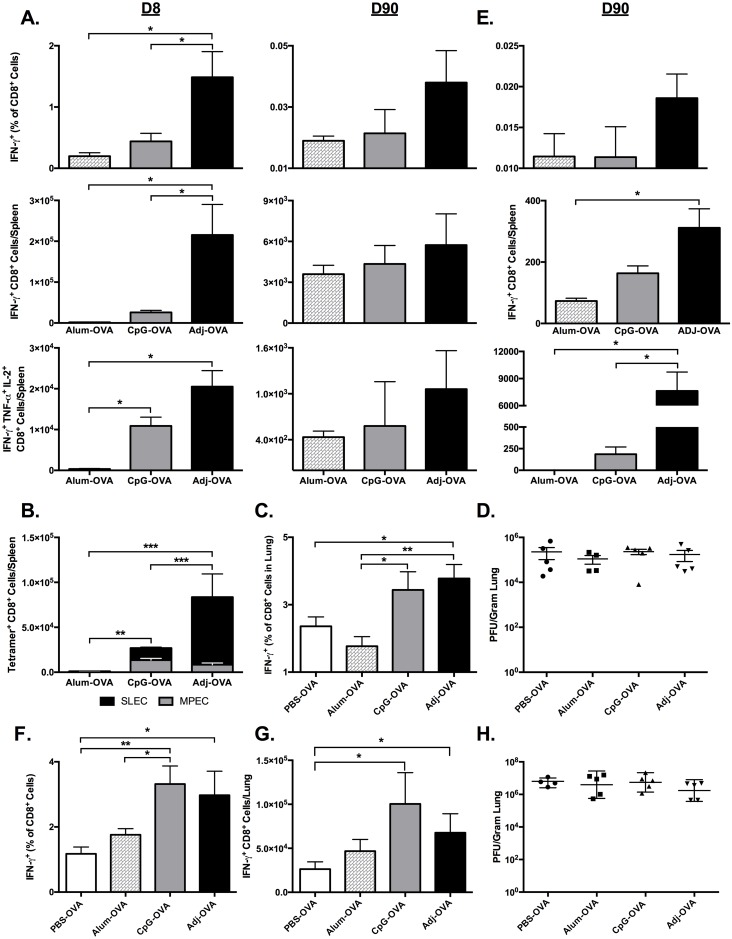
Kinetics of Primary CTL Responses and Viral Control after Subcutaneous Vaccination with OVA. (**A-D**) **Prime-only Vaccination.** C57BL/6 mice were vaccinated by subcutaneous injection of 10 μg ovalbumin in PBS alone or supplemented with 5% Adjuplex or 10 μg CpG. 3–5 mice/group were sacrificed at 8 and 90 days after vaccination. **(A)** The percentages of SIINFEKL-specific IFN-γ^+^ TNF-α^+^ and IL-2^+^ CD8 T cells in spleens were quantified by intracellular cytokine staining at day 8 and 90 after vaccination. (**B**) At day 7 after vaccination, splenocytes were stained with anti-CD8, K^b^/SIINFEKL tetramers, anti-CD127 and anti-KLRG-1. The percentages of short-lived effector cells (SLECs; CD127^LO^/KLRG-1^HI^) and memory precursor effector cells (MPECs; CD127^HI^/KLRG-1^LO^) among tetramer-binding SIINFEKL-specific CD8 T cells were quantified by flow cytometry. Data shows the total numbers of SLECs and MPECs in spleens of vaccinated mice. (**C-D**) Secondary CTL recall responses and lung viral titers after prime-only SQ vaccination and influenza challenge. C57BL/6 mice were vaccinated by SQ injection of 10 μg ovalbumin in PBS alone or supplemented with 5% Adjuplex or 10 μg CpG. 90 days after vaccination, mice were challenged by IN administration of 500 PFU of recombinant influenza A/PR/8/34-OT-I H1N1 expressing the OVA SIINFEKL peptide. 6 days after challenge, 3–5 mice/group were sacrificed and lungs were collected to quantify SIINFEKL-specific CTLs and viral titers. (**C**) Frequency of SIINFEKL-specific IFN-γ^+^ CD8^+^ T cells among all CD8^+^ lung mononuclear cells at 6 days after challenge. (**D**) Lung viral titers expressed as plaque-forming-units (PFU) per gram of lung at 6 days after challenge. * indicates p<0.05, ** indicates p<0.01, *** indicates p<0.001. (**E-H**) **Prime-boost vaccination**. C57BL/6 mice were vaccinated by SQ injection of 10 μg ovalbumin in PBS alone or supplemented with 5% Adjuplex or 10 μg CpG, and boosted 30 days later. (**E**) 3–5 mice/group were sacrificed at 90 days after booster vaccination. Graph shows percentages and/or absolute number of SIINFEKL-specific IFN-γ^+^ TNF-α^+^ and IL-2^+^ CD8 T cells in spleen. (**F-H**) 90 days after vaccination, mice were challenged by IN administration of 500 PFU of recombinant influenza A/PR/8/34-OT-I H1N1 expressing the OVA SIINFEKL peptide. 6 days after challenge, 3–5 mice/group were sacrificed and lungs were collected for quantification of CTLs and viral load. (**F**) Frequency of SIINFEKL-specific IFN-γ^+^ CD8^+^ T cells among all CD8^+^ lung mononuclear cells. (**G**) Absolute numbers SIINFEKL-specific IFN-γ^+^ CD8 T cells in lung. (**H**) Lung viral titers expressed as plaque-forming-units (PFU) per gram of lung. * indicates p<0.05, ** indicates p<0.01. Data is representative of two independent experiments.

Having established that SQ vaccinations with ADJ-OVA can generate strong primary CTL responses, we sought to determine the degree to which the primary CTLs would differentiate into CTL memory. Mice were vaccinated once SQ with 10 μg OVA and either ADJ (5% v/v), Alum (50% v/v), or 10 μg ODN-1826 (CpG). Unlike Alum, CpG has been previously demonstrated to generate CTL responses to OVA and influenza virus proteins.[51–53] At 8 and 90 days after vaccination, spleen and vDLN were collected, and the frequency, number and phenotype of SIINFEKL-specific CTLs were characterized by flow cytometry (Fig 4). The frequency and number of IFN-γ-producing SIINFEKL-specific splenic CTLs generated by ADJ at D8 were significantly (p<0.05) greater than 3-fold compared to Alum, and elevated nearly 3–fold compared to CpG (Fig 4A). Additionally, significant differences (p<0.05) at D8 were observed in the absolute numbers of polyfunctional CTLs co-producing IFN-γ, IL-2, and TNF-α resulting from ADJ as compared to Alum or CpG (Fig 4A). At the peak of the CD8 T-cell response, based on the cell surface expression of KLRG-1 and CD127, effector cells can be classified into two subsets, the short lived effector cells (SLECs; KLRG1^HI^/CD127^LO^) and memory precursor effector cells (MPECs; KLRG1^LO^/CD127^HI^). Data in Fig 4D show that the relative proportions and total numbers of SLECs were higher in the ADJ group, as compared to the Alum and CpG groups. Thus, ADJ appears to promote the differentiation of SLECs, but the total numbers of MPECs were comparable between CpG and ADJ groups (Fig 4B). Next we quantified the frequencies and numbers of SIINFEKL-specific memory CD8 T cells at days 90 after immunization (Fig 4A). Although ADJ stimulated greater expansion of antigen-specific CD8 T cells at day 8 (Fig 4A), there were no differences (P<0.05) in the number of memory CD8 T cells between the groups by D90 (Fig 4A).

In order to assess memory CD8 T cell-dependent protective immunity 90 days post-vaccination, mice from all 3 groups were challenged by intranasal administration of SIINFEKL-expressing recombinant influenza A/PR/8/34-OT-I (PR8-OT-I, Fig 4C and 4D). We chose PR8-OT-I to focus the protection studies on the role of CTLs, as the MHC-I-restricted SIINFEKL peptide was the only shared epitope between the vaccine antigen and challenge virus.[54] At D6 post-infection, the frequency of IFN-γ-producing CTLs in the ADJ group were higher compared to PBS-OVA and ALU-OVA vaccinated mice (Fig 4C). Surprisingly, despite the strong secondary CTL responses in the lungs (Fig 4C), there was no difference in lung viral titers between the groups (Fig 4D). Collectively these data indicated that, a single SQ vaccination with ADJ/CpG/Alum stimulated systemic CTL memory that failed to augment viral control following a mucosal challenge with influenza virus.

Data in Fig 4A–4D suggested that a single parenteral immunization might not elicit sufficient number of memory CTLs to confer protective immunity in the respiratory tract. Therefore, we next sought to determine the effect of prime-boost vaccination on the number of memory CTLs, and the degree to which these cells conferred protective immunity in the lungs. As above, mice were prime-vaccinated SQ with OVA and either ADJ, Alum, or CpG, and the vaccines were repeated 3 weeks later. At 90 days post-boost, the spleen and vDLN were collected, and the frequency and number of SIINFEKL-specific CTLs were characterized by flow cytometry (Fig 4E). At day 90, the numbers of IFN-γ-producing SIINFEKL-specific CD8 T cells in spleen were significantly (p<0.05) greater in the ADJ group, as compared to Alum. Notably, at D90, the number of polyfunctional memory CTLs were significantly (p<0.05) elevated in the ADJ group to approximately 40 times greater than Alum and CpG (Fig 4E).

We next assessed the protective capacity of the memory CTLs generated by prime-boost vaccinations. At 90 days post-boost, mice from all 3 groups were challenged by IN administration of PR8-OT-I (Fig 4F–4H), and 6 days later we quantified CD8 T-cell responses and viral titers in the lung. The number of IFN-γ-producing SIINFEKL-specific CTLs in the lung was greater in the CpG and ADJ groups than Alum and PBS-OVA, however only ADJ was significantly (p<0.05) increased over PBS-OVA (Fig 4G). Surprisingly, despite the presence of high numbers of antigen-specific CD8 T cells in the lungs of ADJ mice, viral loads in the lungs were not significantly different between any treatment groups (Fig 4H). Collectively this indicates that prime-boost SQ ADJ vaccination can enhance the magnitude of systemic antigen-specific polyfunctional memory T cells and the secondary CD8 T-cell recall responses in the lungs, but this does not enhance viral control following influenza virus challenge.

### Intranasal vaccination with Adjuplex generates systemic and respiratory CTL memory, and enhances influenza virus control

The preceding studies demonstrated that prime-boost ADJ expanded systemic CTL memory following prime-boost vaccination, however the expanded CTLs did not enhance viral control following respiratory viral challenge. We next investigated the nature of CTL memory in the lungs following prime-boost SQ or intranasal (IN) vaccination. First we tested whether IN administration of ADJ was tolerated in mice. For vaccination, 3 groups of four 8-10-week old mice were administered intranasally with PBS or PBS plus 10% ADJ. Mice were observed for the first 30 minutes after vaccination, then every 8 hours for 24 hours, then daily until day 7. A subset of mice from each treatment group was boosted with identical vaccines 21 days after priming, and observed daily for 21 more days. No change in behavior or appetite was observed, and the mice did not lose weight during the first 7 days. Lungs were collected from PBS- and ADJ-vaccinated mice at 1 and 7 days after prime, and 21 days after boost. Histological evaluation of the lungs found no significant abnormalities at any time point (S1 Fig). Thus, ADJ alone did not lead to pulmonary lesions or discernible distress or disease following IN administration to mice.

**Fig 5:**
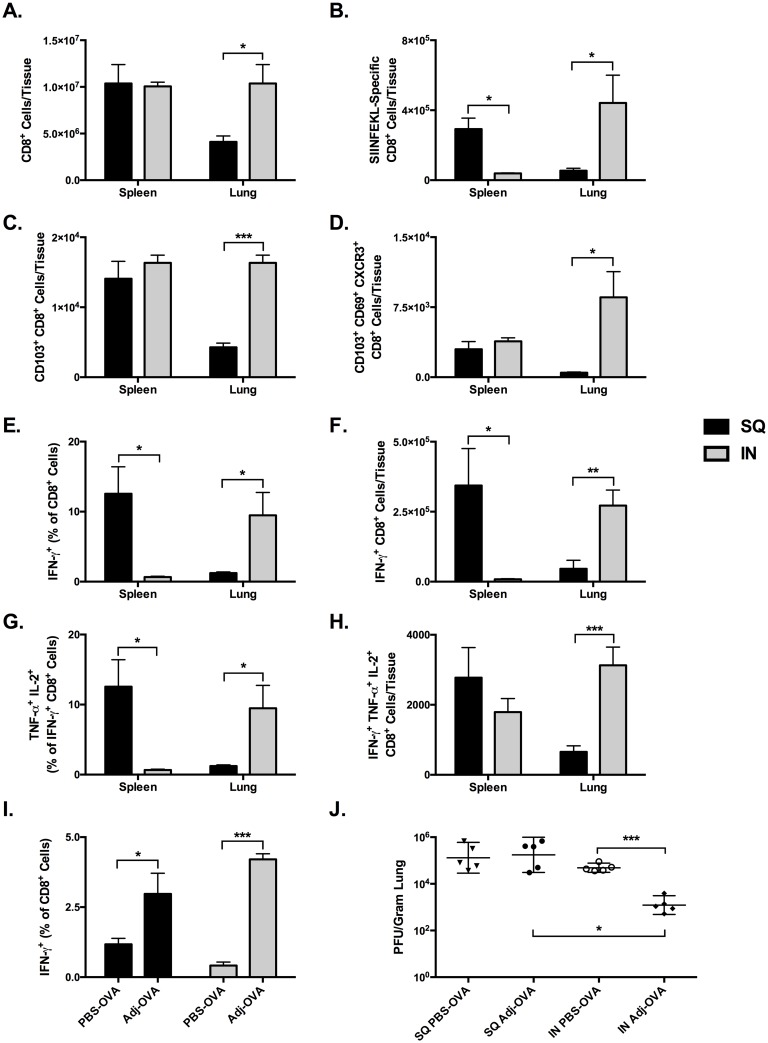
Comparison of CTL Memory and Recall Responses in Lung and Spleen after SQ or IN Prime-boost Vaccination with OVA. C57BL/6 mice were immunized intranasally with 10 μg of OVA in 50 μl PBS with 5% ADJ (SQ) or 10% ADJ (IN) twice at 3 week intervals. Lung and spleen were collected 3 weeks after the second vaccine. (**A-F)** Lung cells and splenocytes were stained with anti-CD8, anti-CD44, anti-CD62L, anti-CD103, anti-CD69, anti-CXCR3 and K^b^/SIINFEKL tetramers; cells were acquired on a flow cytometer. (**A**) Absolute numbers of CD8 T cells in the lung and spleen. (**B**) Absolute numbers of SIINFEKL-specific tetramer-binding CD8 T cells in the lung and spleen. (**C**) CD103^+^ SIINFEKL-specific CD8 T cells in lung and spleen. (**D**) Absolute numbers of SIINFEKL-specific tetramer-binding resident memory-like CD103^+^CD69^+^CXCR3^+^ CD8 T cells in the lung and spleen. (**E-H**) Lung cells and splenocytes were stimulated with the SIINFEKL peptide and the percentages of IFN-γ^+^ IL-2^+^ TNF-α^+^ CD8 T cells were quantified by flow cytometry. (**E**) Frequency of SIINFEKL-specific IFN-γ^+^ CTLs among all CD8^+^ cells in lungs and spleen. (**F)** Absolute numbers of SIINFEKL-specific IFN-γ^+^ CTLs. (**G**) Frequency of SIINFEKL-specific IFN-γ^+^ IL-2^+^ TNF-α^+^ CTLs among all CD8^+^ cells. (**H)** Absolute numbers SIINFEKL-specific IFN-γ^+^ IL-2^+^ TNF-α^+^ CTLs. (**I-J)** C57BL/6 mice were immunized intranasally with 10 μg of OVA in 50 μl PBS with 5% ADJ (SQ) or 10% ADJ (IN) twice at 3 week intervals. 3 weeks after the second immunization, mice were challenged by intranasal administration of 500 PFU of recombinant influenza A/PR/8/34-OT-I H1N1 expressing the OVA SIINFEKL peptide. 6 days after challenge 5 mice/group were sacrificed and lungs were collected for quantification of CTLs and viral titration. (**I)** Frequency of SIINFEKL-specific IFN-γ^+^ CD8^+^ T cells among all CD8^+^ lung mononuclear cells. (**J)** Lung viral titers expressed as plaque-forming-units (PFU) per gram of lung. * indicates p<0.05, ** indicates p<0.01, *** indicates p<0.001. Data is representative of two experiments.

To determine the effect of SQ and IN vaccinations on CTL memory in the lungs, we vaccinated separate groups of mice via SQ or IN routes. The SQ-vaccinated mice received 10 μg OVA in PBS + 5% ADJ while the IN-vaccinated group received 10 μg OVA in + 10% ADJ, and all vaccines were boosted 21 days later. At 21 days post-boost, the frequency, number and phenotype of SIINFEKL-specific CTLs in lung and spleen were characterized by flow cytometry (Fig 5). IN vaccination resulted in a significant (p<0.05) 2.5-fold increase in the absolute number of CD8^+^ lymphocytes in the lung compared to SQ, while no differences in number of CD8^+^ lymphocytes were observed in the spleen (Fig 5A). The number of SIINFEKL-tetramer^+^ CTLs in the lungs in the IN group was 8-fold larger than SQ (p<0.05), while the number of tetramer+ cells in the spleen was more than 7-fold larger following SQ vaccination (p<0.01, Fig 5B). In the lung, the absolute number of CD103^+^ CTLs was 4 times larger in the IN group than in the SQ group (p<0.001), however there were no differences between the two groups in the numbers of CD103^+^ CTLs in the spleen (Fig 5C). Notably, IN vaccination resulted in a 17-fold greater number of CD103^+^ CD69^+^ CXCR3^+^ resident memory-like CTLs (T_RM_) compared to SQ (p<0.05, Fig 5D). In contrast, intermediate numbers of T_RM_-like cells that were not significantly different were found in the spleen in both groups. In summary, compared to prime-boost SQ-ADJ vaccination, IN vaccination with ADJ generated substantial populations of CD103^+^ CD69^+^ CXCR3^+^ T_RM_-like CTLs in the lungs. By comparison, SQ-ADJ vaccine elicited larger frequencies and numbers of memory CTLs in the spleen.

Next, we compared functional cytokine-producing CD8 T cells in the lung and spleen of vaccinated mice. In the lung, following IN vaccination, the frequency of IFN-γ-producing SIINFEKL-specific CTLs was 7 fold greater than SQ (p<0.05), while the frequency in the spleen was nearly four-fold higher in the SQ group compared to the IN group (p<0.05, Fig 5E). Similarly, the absolute number of IFN-γ-producing SIINFEKL-specific CTLs in the lungs of IN group was four-fold greater than SQ (p<0.01), while the numbers in the spleen was more than four-fold greater in the SQ group compared to the IN group (Fig 5F). Differences were more pronounced in the polyfunctional CTLs co-producing IFN-γ, IL-2, and TNF-α. The frequency of these triple cytokine-producing CTLs in the lung in the IN group was four-fold greater than the SQ group (p<0.05, Fig 5G), and translated into 2 times as many triple cytokine^+^ cells (p<0.001, Fig 5H). Conversely, the frequencies of triple cytokine^+^ cells in the spleen following SQ vaccination were 5 fold greater than IN (p<0.05), however the absolute number of cells was not significantly different. Remarkably, when taken together, the data in Fig 5 indicate that IN and SQ vaccinations elicit mucosal and systemic CTL memory respectively.

We next compared the protective recall capacity of memory CD8 T cells between IN and SQ vaccinees. Mice were prime-boost vaccinated via SQ or IN routes as above. At 21 days post-boost, mice were infected by IN administration of PR8-OT-I, and 6 days later we quantified secondary CD8 T-cell responses and viral titers in the lungs. The number of CD8^+^ T lymphocytes in the spleen and lungs was the same whether mice were vaccinated with ADJ via the SQ or IN route (S2 Fig). Surprisingly, we found that IFN-γ^+^ CTL recall responses in the lungs were of similar magnitude in both the ADJ SQ- and IN-vaccinated groups, and these responses were 3-fold or more greater than SQ without ADJ (p<0.05) and IN without ADJ (p<0.001) (Fig 5I). This demonstrated a clear adjuvant effect in the ADJ groups, and strong recall responses regardless of administration route. Importantly however, despite the similarities in the magnitude of the CTL recall responses, only immunity generated in the ADJ-IN group protected against virus, decreasing viral titers by nearly 100 fold (Fig 5J).

To address whether improved viral control in intranasally vaccinated mice was linked to altered distribution of secondary CTLs in the lung airways and parenchyma, we vaccinated groups of mice with ADJ-OVA via SQ or IN routes. At 21 days after vaccination, mice were challenged with PR8-OT-I. Six days after challenge, we quantified SIINFEKL-specific secondary CTLs in airways (bronco-alveolar lavage; BAL) and lungs. Data in S3 Fig show that there were ~2-fold more SIINFEKL-specific CTLs in the BAL of IN vaccinated mice than in SQ vaccinated mice. Thus, enhanced influenza viral control following IN vaccination was associated with increased numbers of CTLs in the airways.

**Fig 6:**
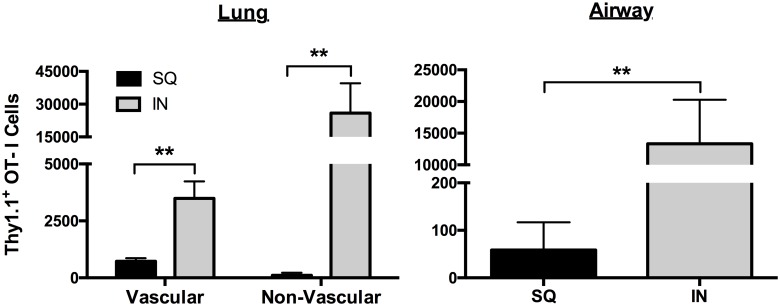
Memory CTLs in the lung vasculature, parenchyma and airways following IN vaccination with ADJ-OVA. Thy1.1^+ve^ naïve OT-I TCR transgenic CD8 T cells were adoptively transferred into C57BL/6 mice. Twenty-four hours after cell transfer, mice were vaccinated with ADJ-OVA by the SQ or IN routes. Three weeks after the first vaccination, mice received a booster vaccination. Twenty-one days after the booster vaccination, OT-I cells in vasculature, parenchyma and airways were quantified by flow cytometry. Vascular OT-I CD8 T cells were identified by intravascular staining; mice were infused intravenously with anti-CD8β antibodies 5 minutes before euthanasia. Following euthanasia, cells in the BAL and lungs were stained with anti-Thy1.1, anti-CD8α, anti-CD103, anti-CXCR3 and anti-CD69 antibodies. Vascular OT-I cells were identified based on positivity for both CD8α and CD8β and non-vascular cells were positive only for CD8α. Data is from one of two independent experiments; ** indicates p<0.01.

Data in Fig 5 show that IN vaccination with ADJ-OVA elicited markedly greater number of T_RM_-like CD8 T cells in the lungs. In addition to the induction of T_RM_, strategic positioning of memory T cells in the tissues is of critical importance in engendering protective immunity. Using intravascular staining technique in combination with adoptive transfer of OVA-specific OT-I CD8 T cells, we assessed whether SQ and IN vaccination differed in terms of the localization of memory T cells in the lung vasculature, parenchyma or airways. As illustrated in Fig 6, IN vaccination with ADJ-OVA elicited a significantly (p<0.01) greater number of vascular and non-vascular memory OT-I cells in the lungs, as compared to those in SQ vaccinated mice. Memory OT-I CD8 T cells were barely detectable in the airways of SQ ADJ-OVA mice (Fig 6). In striking contrast, IN vaccination with ADJ-OVA potently elicited a significantly (p<0.01) greater number of memory T cells in the airways (Fig 6). Thus, IN ADJ-OVA vaccination elicits strong CD8 T cell memory in lung vasculature, parenchyma and airways.

### Intranasal vaccination with Adjuplex-adjuvanted inactivated influenza protects against heterosubtypic challenge

We next evaluated whether protection afforded by IN ADJ vaccination with the model antigen OVA, illustrated above, would extend to pathogen-associated antigens. We repeated the IN prime-boost vaccine protocol described above, but replaced OVA with varying concentrations of beta-propiolactone (BPL)-inactivated influenza A virus. The inactivated influenza virus, PR8-Tex H3N2, is a reverse-genetics-derived virus containing HA and NA genes from A/Texas/50/2012 H3N2 with the structural genes from A/PR/8/34 H1N1 influenza virus. Typically, commercial influenza virus vaccines are standardized based on the amount of HA. Therefore, viral protein concentrations in our vaccine preparations were normalized based on the amount of HA. It should be noted that the vaccine preparation contains other influenza viral proteins including nucleoprotein (NP), polymerase acidic protein (PA), neuraminidase and matrix proteins, in addition to HA. The inactivated preparation of PR8-Tex virus containing different concentrations of HA1 protein with and without 10% ADJ was used to immunize mice by the IN route; mice vaccinated with ADJ alone served as negative control. Twenty-one days after the initial vaccination, the vaccines were repeated to complete the prime-boost protocol. At 21 days post-boost, mice were challenged by IN inoculation with heterosubtypic influenza A/PR/8/34 H1N1. Six days after infection, mice were euthanized and lungs were collected to analyze virus-specific CTL responses and viral titers.

**Fig 7:**
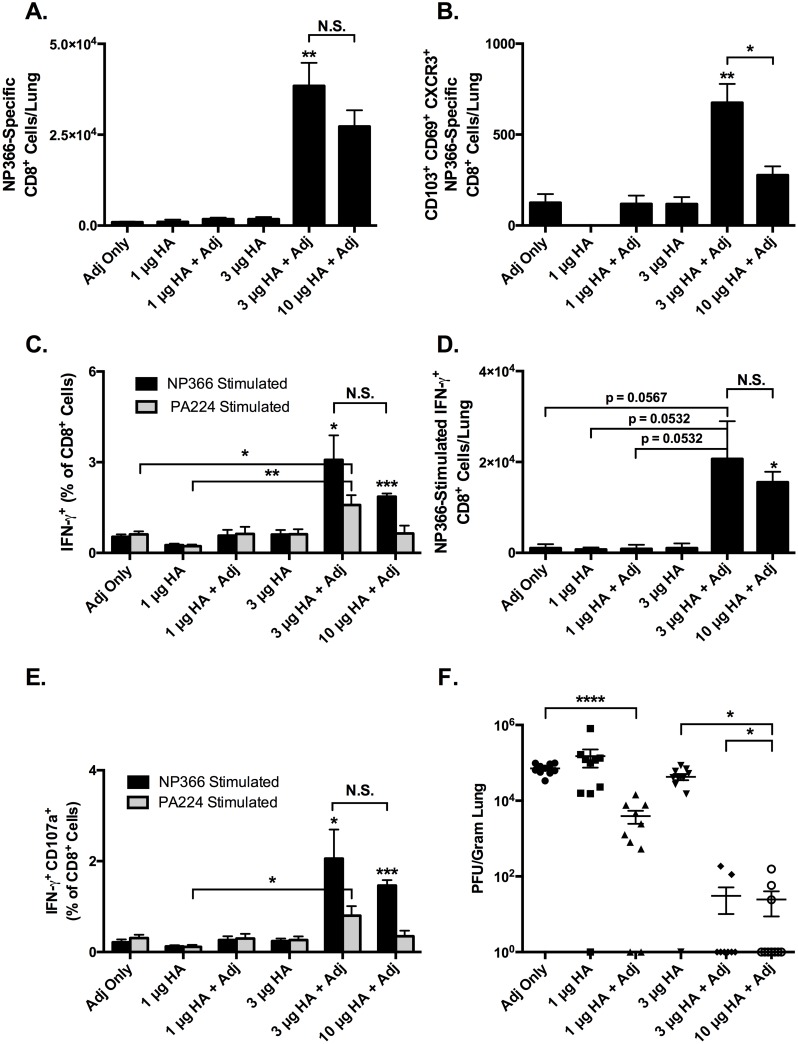
CTL Recall Responses and Protective Heterosubtypic Immunity to Influenza Virus after Prime-boost IN Vaccination. C57BL/6 mice were immunized intranasally with 1, 3, or 10 μg of HA-equivalent of BPL-inactivated influenza strain PR8xTexas/H3N2 in 50 μl PBS with or without 10% ADJ. Note that the vaccine preparation also contains other viral proteins including NP, PA, neuraminidase and matrix proteins. Vaccines were repeated 3 weeks later, and 3 weeks after the second immunization, mice were challenged by IN administration of 200 PFU of influenza A/PR/8/34/H1N1. 6 days after challenge 5 mice/group were sacrificed and lungs were collected for CTL analysis and viral titration. D^b^/NP366-specific CD8 T cells in lung were quantified by MHC I tetramers or intracellular cytokine staining for IFN-γ. (**A)** Absolute numbers SIINFEKL-specific IFN-γ^+^ CTLs in lung. (**B)** Absolute numbers of CD103^+^ CD69^+^ CXCR3^+^ NP366-tetramer^+^ CTLs in lungs. (**C)** Frequencies of IFN-γ-producing CTLs specific for influenza NP366 and PA224 epitopes among all CD8^+^ cells in the lungs. (**D**) Total number of IFN-γ-producing CTLs specific for influenza NP366 epitope in the lungs. (**E)** Frequencies of CTLs double positive for IFN-γ and CD107a (LAMP-1) among all CD8^+^ cells in the lungs. (**F**) Lung viral titers expressed as plaque-forming-units (PFU) per gram of lung. * indicates p<0.05, ** indicates p<0.01, **** indicates p<0.0001. In panels A-E only, asterisks over a specific bar indicate significance over other groups, unless otherwise indicated. ** indicates significant difference of 3μg HA+ ADJ group with other groups excluding 10μg HA + ADJ. T-cell data are representative of two independent experiments. Viral titer data is pooled from two separate experiments.

The number and phenotype of CTLs specific for the influenza NP epitope ASNENMETM (NP366) were assessed by using MHC I tetramers and ICCS for IFN-γ (Fig 7). The absolute number of NP366-tetramer^+^ cells in the 3 μg and 10 μg HA+ADJ groups was 10–25 times larger than the other groups (all p<0.05, Fig 7A). In contrast, the absolute number of tetramer^+^ cells generated by 1 μg HA, 1 μg HA+ADJ, and 3 μg HA were no different than ADJ alone (Fig 7A). However, 3 μg HA+ADJ yielded a 20-fold increase in the number of tetramer^+^ cells over 3 μg HA alone, and was similar to 10 μg HA+ADJ. Likewise, the absolute number of CD103^+^ CD69^+^ CXCR3^+^ CTLs in the lungs was 2–3 times larger in the 3 μg and 10 μg HA+ADJ groups (Fig 7B). Thus, ADJ potently enhanced the immunogenicity of an inactivated influenza A virus vaccine and elicited potent CTL recall responses to experimental heterosubtypic influenza A virus infection.

We also found dose-dependent increases in the magnitude of the CTL responses as measured by frequency and number of IFN-γ^+^ cells. Fig 7C compares the frequencies of NP366- and PA224-specific IFN-γ-producing CTLs for each treatment group. Consistent with the increase in the numbers of tetramer-binding virus-specific CD8 T cells, the recall responses of IFN-γ-producing CD8 T cells were markedly enhanced in the 3 μg HA+ADJ and 10 μg HA+ADJ groups (Fig 7C and 7D). Additionally, the majority of the NP366- and PA224-specific IFN-γ^+^ cells were also positive for CD107a (LAMP-1), a marker of degranulation and indicator of cytolytic capacity (Fig 7E). The increased magnitude of the CTL response at higher doses of HA+ADJ was reflected in markedly decreased lung viral titers (Fig 7F). Viral titers at 1 μg HA and 3 μg HA were similar to ADJ alone, while the group given 1 μg HA+ADJ had a 1 log decrease than ADJ alone (p<0.0001), and the groups given 3μg HA+ADJ and 10 μg HA+ADJ were similar with nearly 3-log decrease in titer compared to ADJ alone (p<0.0001) and 2-log decrease compared to 1 μg HA+ADJ (p<0.05). Virus was not detected in 1 mouse each from the 1μg HA, 1 μg HA+ADJ and 3 μg HA groups, while virus was only detected in 2 of 10 mice in the 3 μg HA+ADJ and 10 μg HA+ADJ groups. Thus, the adjuvanted vaccines provided significant protection against heterosubtypic influenza viral challenge. In summary, the addition of ADJ to the vaccines resulted in enhanced viral control following influenza virus challenge, and doses of 3 and 10 μg HA+ADJ resulted in viral titers below the level of detection in 8 of 10 and 7 of 10 mice respectively. This protection correlated with increases in the number of NP366- and PA224-specific CTLs with increased IFN-γ-production, and higher expression levels of CD107a, CD69, and CD103.

### MyD88-deficiency does not affect the magnitude of primary CTL responses to Adjuplex vaccines but alters effector differentiation

We next investigated possible mechanisms underlying the CTL-activating effects of ADJ. MyD88 is a common adapter protein in the signaling pathway for all toll-like receptors except TLR3. MyD88 is important for the activation of DCs and other innate cells, and the production of inflammatory cytokines such as IL-18 and IL-1β. Furthermore, MyD88 deficiency has adverse effects on T-cell proliferation, and T_H_1 differentiation and effector function.[55] Cohorts of WT and MyD88^-/-^ mice were vaccinated SQ with 10 μg OVA and 5% ADJ in 50 μl PBS. On day 8 after vaccination, spleens were collected and the frequency, number and phenotype of SIINFEKL-specific CTLs generated by the prime vaccination were characterized by flow cytometry.

**Fig 8:**
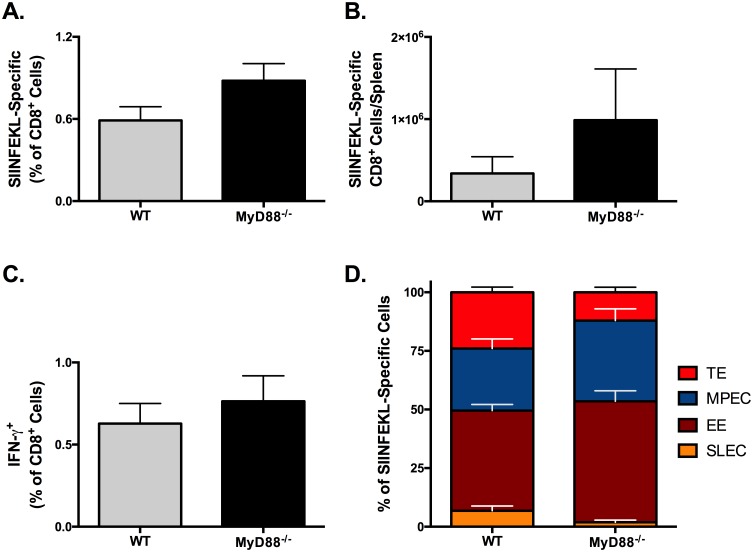
MyD88-deficiency Does Not Affect the Magnitude of Primary CTL Responses to SQ Adjuplex Vaccines, but Does Alter Memory Differentiation. Cohorts of wild type (WT) and MyD88^-/-^ mice were vaccinated SQ with 10 μg OVA and 5% ADJ in 50 μl PBS. On day 8 after vaccination, spleens were collected and the frequency, number and phenotype of SIINFEKL-specific CTLs were characterized by flow cytometry. (**A)** Frequency of SIINFEKL-tetramer^+^ CTLs cells in the spleen among all CD8^+^ lymphocytes. (**B)** Absolute numbers of SIINFEKL-tetramer^+^ CTLs in the spleen**.** (**C**) Frequency of SIINFEKL-specific IFN-γ^+^ CTLs cells in the spleen among all CD8^+^ lymphocytes. (**D)** Percentages of SLECs (KLRG-1^HI^CD127^LO^), MPECs (KLRG-1^LO^CD127^HI^), transition effectors (TEs; KLRG-1^HI^CD127^HI^) and early effectors (EEs; KLRG-1^LO^CD127^LO^) among SIINFEKL-specific CD8 T cells in spleen. Data are from 3–5 mice/group and representative of two independent experiments. * indicates p<0.05.

The frequency of SIINFEKL-specific CTLs (Fig 8A) in spleen of MyD88^-/-^ mice was similar to those in WT mice. However, consequent to increased splenic cellularity in vaccinated MyD88^-/-^ mice, the total number of SIINFEKL-specific CD8 T cells in these mice was higher than in WT mice (Fig 8B). The percentages of SIINFEKL-specific CD8 T cells producing IFN-γ (Fig 8C) in WT and MyD88^-/-^ mice were nearly identical. We also analyzed whether MyD88 deficiency affected the differentiation of effector subsets in the spleen. Strikingly, the relative proportions of transition effectors (TE; KLRG-1^HI^CD127^HI^) and SLECs among SIINFEKL-specific CD8 T cells were decreased in the MyD88^-/-^ mice (Fig 8D). Collectively these data demonstrated that MyD88 deficiency has little effect on CTL priming or the magnitude of initial responses to SQ ADJ vaccination, however it skewed the response towards the less terminally differentiated KLRG-1^LO^ phenotype. These data indicate that ADJ activation of CTLs is MyD88-independent, however MyD88 does play a role in CTL differentiation, as previously reported.[55]

### Effect of Adjuplex on inflammatory cells and DCs in vaccine draining lymph nodes and lungs

**Fig 9:**
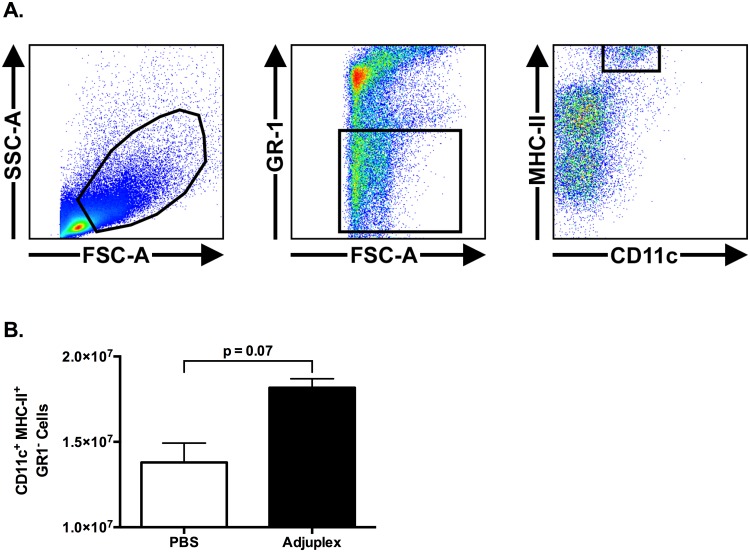
Effect of Adjuplex on Conventional Dendritic Cells in the Vaccine Draining Lymph Node After Subcutaneous Vaccination. Mice were vaccinated by SQ injection with 10 μg OVA in 50 μl of PBS with and without either 5% ADJ. The vaccine draining lymph nodes were collected at 48 hours after vaccination and analyzed by flow cytometry. (**A)** Gating strategy for CD11c^+^/GR1^-^/MHC-II^HI^ cells among all cells with forward scatter greater than, and side scatter equal to or greater than lymphocytes. (**B)** Number of CD11c^+^/GR1^-^/MHC-II^HI^ cells in DLNs. Data shown are representative of two independent experiments.

For our initial investigation into the effect of ADJ on DC populations in vivo, mice were vaccinated by SQ injection with 10 μg OVA in 50 μl of PBS with and without either 5% ADJ. The vDLN were collected at 48 hours after vaccination and analyzed by flow cytometry. Activated conventional DCs were identified as CD11c^+^ GR-1^-^ MHC-II^HI^ cells with FSc/SSc parameters greater than the lymphocyte gate (Fig 9A). At 48h, there was an increase in the number of activated conventional DCs in vaccine draining lymph nodes (vDLNs) of ADJ mice, as compared to those in PBS mice (Fig 9B). These data suggested that ADJ increases conventional DCs in the draining lymph nodes.

**Fig 10:**
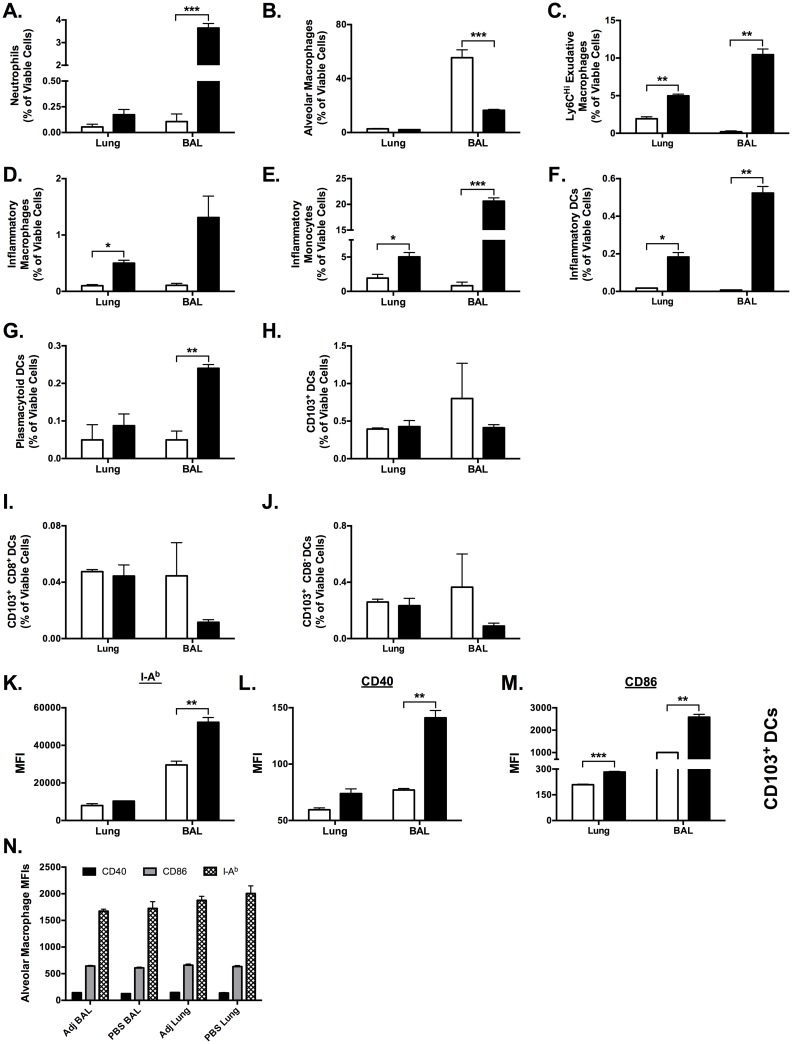
Intranasal Adjuplex Administration Rapidly Alters Inflammatory Cell Populations in Bronchoalveoloar Lavage Fluid and Lung Tissue. Mice were vaccinated by IN inoculation of 50 μl PBS with and without 10% ADJ, and the bronchoalveolar lavage (BAL) fluid and lung were collected 24 hours later and analyzed by flow cytometry. Absolute cell counts in BAL and lung were not significantly different. Populations of individual cells were identified by the gating paradigm described in S5 Fig. (**A-J**) Percentages of the indicated cell populations (neutrophils, alveolar macrophages, Ly6c^HI^ exudative macrophages, inflammatory macrophages, inflammatory monocytes, inflammatory DCs, plasmacytoid DCs, CD103^+^ DCs, CD103^+^DCs, CD103^+^CD8^+^ DCs and CD103^+^CD8^-^ DCs) are expressed and frequencies of all viable cells. (**K-M)** Median fluorescent intensity (MFI) of MHC-II (I-A^b^), CD40, and CD86 expression on CD103^+^ DCs. (**N**) MFI of MHC-II (I-A^b^), CD40, and CD86 expression on alveolar macrophages. * indicates p<0.05, ** indicates p<0.01, *** indicates p<0.001. Data represents two independent experiments.

We next investigated the effect of ADJ on recruitment and activation of inflammatory cells to the airways and lungs following IN vaccination. Mice were vaccinated by IN inoculation of 50μl PBS with and without 10% ADJ, and bronco-alveolar lavage (BAL) fluid and lungs were collected 24 hours later. At 24 h after vaccination, ADJ induced significant alterations in multiple innate cell populations in the lung airways (BAL fluid), and exerted modest effects on select populations in the lung (Fig 10). Absolute cell counts in the BAL fluid and lungs were not different (p<0.05) between groups (S4 Fig). In the BAL, neutrophils accounted for 4% of cells in the ADJ group, but were barely detectable in the PBS group (p<0.001, Fig 10A). Conversely, alveolar macrophages comprised >50% of BAL cells in the PBS group, but only 15% in the ADJ group (p<0.01, Fig 10B). Notably, the percentages of exudative macrophages (Fig 10C), inflammatory monocytes (Fig 10E) and inflammatory DCs (Fig 10F) in the BAL and lungs of ADJ mice were significantly (p<0.05) greater than in PBS mice. ADJ did not alter the percentages of CD103^+^ (Fig 10H–10J) or the CD103^-^ DCs. However, the expressions of MHC-II (Fig 10K), CD40 (Fig 10L) and CD86 (Fig 10M) on CD103^+^ DCs in the BAL of ADJ mice were significantly (p<0.05) higher than in the PBS mice.

Expression levels of cell-surface surface molecules MHC-II (I-A^b^), CD40, and CD86 were used to evaluate the activation of antigen-presenting cells in the BAL fluid and lung. Strikingly, the CD103^+^ DCs in the ADJ BAL had significantly higher expression of MHC-II, CD40, and CD86 than PBS alone (all p<0.01, Fig 10K–10M). Expression of MHC-II, CD40, and CD86 on alveolar macrophages was unaffected, and levels were similar in the in BAL fluid and lung (Fig 10N). Taken together, ADJ significantly augmented in the lung airways (BAL), the activation of CD103^+^ DCs, which are known to play a prominent role in cross presentation of exogenous antigens to CD8 T cells.[56]

### Adjuplex activates DC2.4 cells and alters antigen uptake, processing, and intracellular localization

**Fig 11:**
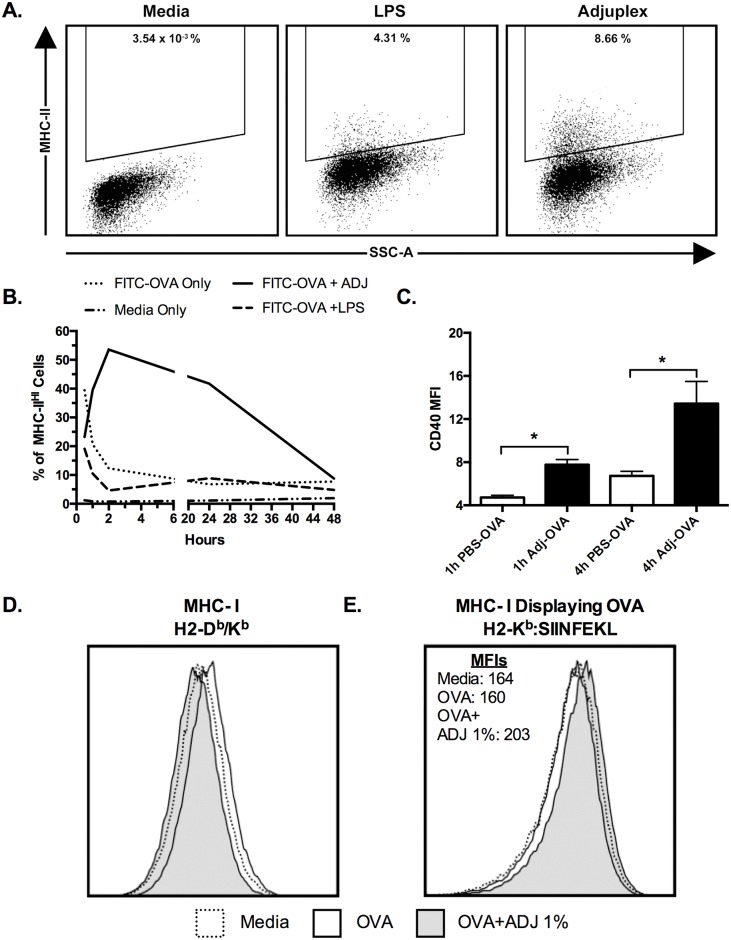
Adjuplex Alters Antigen Presentation and Activation of DC2.4 cells. (**A-B)** DC2.4 cells were cultured with 200 ug/ml FITC-OVA alone or supplemented with 1% ADJ or 5 μg /ml LPS. After 30 minutes, the treatment media was aspirated, washed, and maintained in growth media. The cells were harvested at 0.5, 1, 1.5, 2, 24 and hours and processed for flow cytometry. Panel A shows the gating strategy for MHCII^HI^ DC2.4 cells. Graph in **(B)** shows the kinetics of MHC-II expression on all DC2.4 cells as measured by the MFI of anti-I-A^b^ (MHC-II) antibodies. **(C**) DC2.4 cells were cultured in a 96-well plate in media containing 200 μg/ml FITC-OVA with or without 1% ADJ for 1 or 4 hours. Magnitude of DC2.4 cell activation as measured by the MFI of CD40 on all DC2.4 cells. (**D)** MHC-I expression on DC2.4 cells 24h after pulse, as characterized by fluorescent labeling of MHC-I with anti-H2-D^b^/K^b^ antibodies. (**E)** Expression of the MHC-I allele H2-K^b^ bearing SIINFEKL peptide (H2-K^b^:SIINFEKL) at 24h after pulse, as characterized by fluorescent labeling by antibodies specific for the K^b^:SIINFEKL-complex**)**. Error bars = SEM, * = p<0.05. Data are representative of at least two independent experiments.

In order to investigate the effects of ADJ on antigen uptake, processing and presentation by DCs, we next pursued in vitro studies using DC2.4 cells, an immortalized murine DC-like cell line. DC2.4 cells are considered to be similar to immature dendritic cells in vivo, and have been used extensively in studies of antigen uptake and processing, and are capable of presenting antigen on class I and class II MHC molecules, and priming naïve T cells.[57–59] For our initial studies, we cultured DC2.4 cells in growth media containing 200 μg/ml FITC-OVA with or without 1% ADJ for 1 or 4 hours. We evaluated the expression of cell-surface markers CD11b, CD11c, CD80, CD86, CD40, and IFN-γR1 at both time points. ADJ treatment did not alter the expression levels of CD80/CD86 but increased the expression of CD40 (Fig 11C). CD11c and IFN-γR1 were not detectable at any time point. In a parallel experiment, we pulsed DC2.4 cells with OVA or OVA with ADJ/LPS for 30 minutes. At different time points after the pulse, we quantified MHC II expression on DC2.4 cells by flow cytometry. As shown in Fig 11A and 11B, MHC-II expression was detected in all treatment groups at 30 minutes, with 40% of cells in the LPS group, and 20–25% of cells in the LPS-OVA and ADJ-OVA groups, while it was expressed by less than 2% of cells in the media-only group (Fig 11B). Over the first 2 hours, frequency of MHC-II^+^ cells rapidly decreased to less than 15% in the LPS-OVA and LPS-ONLY groups, however the frequency in the ADJ-OVA group rapidly increased to over 50% of cells at 2 hours, remained greater than 40% at 24h, and was slightly elevated over the other groups at 48h. Thus, data in Fig 11A–11C suggested that ADJ is a potent activator of DC2.4 cells.

Because of the large disparity in MHC-II expression at 24h after pulse, we repeated the pulse-chase experiment with only media, FITC-OVA, and FITC-OVA-ADJ, and looked at the overall expression of class I and II MHC molecules, and specifically looked for differential expression of MHC-I molecules bearing SIIFNKEL peptide at 24h after pulse. Expression of all MHC-I alleles, as evaluated with pan-reactive antibodies against H2-D^b^ and H2-K^b^, was not significantly different between groups (p>0.05) (Fig 11D). Expression of the SIINFKEKL-bearing MHC-I molecules was evaluated with antibodies specifically targeting the H2-K^b^/SIINFEKL complex. Interestingly, there were no differences between media and the OVA-only groups, however the ADJ-OVA group exhibited a modest but reproducible increase in K^b^/SIINFEKL expression (Fig 11E). These data suggested that ADJ might enhance antigen presentation by DCs to CD8 T cells.

**Fig 12:**
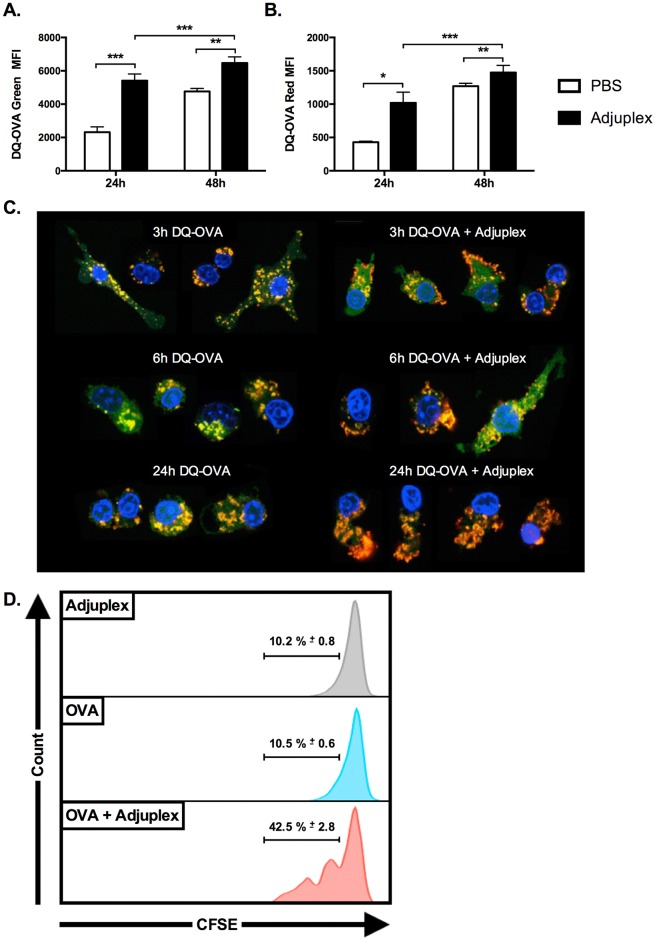
Adjuplex Alters Antigen Processing and Intracellular Localization by DC2.4 cells. (**A-B**) DC2.4 cells were cultured in media containing 200 μg/ml DQ-OVA alone or supplemented with 1% ADJ. After 30 minutes, the treatment media was aspirated; the cells were washed twice with growth media, and then maintained in growth media. The cells were harvested at 24 and 48h and processed for flow cytometry. DQ-OVA is a self-quenched dye that exhibits green fluorescence after proteolytic cleavage, and red secondary or excimer fluorescence upon aggregation of the processed green fluorescent molecules. (**A)** Magnitude of DQ-OVA primary green fluorescence with and without ADJ treatment, as determined by MFI. (**B)** Magnitude of DQ-OVA excimer red fluorescence with and without ADJ treatment, as determined by MFI. * indicates p<0.05, ** indicates p<0.01, *** indicates p<0.001. (**C)** Adjuplex alters the intracellular localization of antigen in DC2.4 cells. DC2.4 cells were plated on coverslip glasses within 24-well plates and grown overnight in growth medium. 24 hours later the medium was replaced with 200 μg/ml DQ-OVA with and without ADJ. After 30 minutes, the media was aspirated, cells were washed twice with growth media, and then maintained in growth media. At the indicated intervals, coverslips were washed twice in in PBS then fixed in 1% PFA for 10 minutes, permeabilized with 0.1% saponin, and incubated with DAPI 1 μg/ml for 5 minutes. Cells were imaged on a Leica confocal laser scanning microscope. Green = DQ-OVA primary fluorescence, red/orange = DQ-OVA excimer fluorescence, yellow = merge, blue = nucleus. Column on the left shows images at different time points after pulse with DQ-OVA only. Column on the right shows images of DC2.4 cells pulsed with DQ-OVA + Adjuplex. Images are composites from 2 different experiments and representative of 30 cells per group. (**D**) DC2.4 cells were exposed to ADJ, OVA or ADJ+OVA for 30 minutes. DC2.4 cells were washed and cultured with CFSE-labeled naïve OT-I CD8 T cells for 72 hrs. Cells were acquired on a BD LSR Fortessa flow cytometer, and CFSE fluorescence of OT-I cells was quantified by flow cytometry. Histograms are gated on OT-I CD8 T cells and the numbers are the percentages of gated cells that have reduced levels of CFSE fluorescence, indicative of proliferation. Data is derived from one of two independent experiments.

DQ-OVA is OVA labeled with a self-quenched BODIPY-FL dye. The primary green fluorescence of DQ-OVA only occurs after proteolytic cleavage, indicating active antigen processing. A secondary red florescence can be detected if the degradation products form adequately large aggregates, or excimers, within the cells. To determine whether ADJ affected antigen processing, we pulsed DC2.4 cells with DQ-OVA with and without ADJ for 30 minutes, and evaluated the primary and excimer fluorescence by flow cytometry 24 and 48h later. As illustrated in Fig 12A and 12B, the magnitude of primary and excimer fluorescence was significantly increased from 24 to 48h in both groups. Both primary fluorescence and excimer fluorescence in the ADJ group at 24h were twice the magnitude of the PBS group, though differences between groups were smaller at 48h. These data along with data in Fig 11 suggested that ADJ potently increases or accelerates antigen processing and presentation by DC2.4 cells.

To further investigate antigen processing and intracellular localization in DC2.4 cells, we repeated the DQ-OVA pulse-chase experiment and analyzed the cells by confocal microscopy at 3, 6, and 24h post-pulse. As illustrated in Fig 12C, the amount of primary and excimer fluorescence and the intracellular localization of the antigen were dynamically altered by the presence of ADJ. In the absence of ADJ, DQ-OVA was primarily localized to punctate cytoplasmic aggregates randomly distributed throughout the cells with localization of some larger aggregates in perinuclear regions in some cells (Fig 12C). The majority of the punctate aggregates exhibited green primary florescence with the larger perinuclear aggregates exhibiting more mixed (yellow) and excimer fluorescence (orange-red). In contrast, the ADJ-treated cells exhibit a homogenous to amorphous distribution of green primary fluorescence throughout the cytoplasm, with fewer to larger globoid aggregates tending toward mixed yellow fluorescence, and striking bright orange, irregularly segmental bands of fluorescence subtending the cell membrane (Fig 12C). By 6 hours, the green punctate aggregates in PBS group were larger and slightly less well defined, and were accompanied by slightly increased in amorphous green background fluorescence (Fig 12C), while the ADJ group generally exhibited a mix of membrane-associated orange linear to irregular orange aggregates (Fig 12C). The PBS group exhibited minor changes by 24 hours, primarily characterized by an increase in homogeneous green cytoplasmic fluorescence, and larger and less distinct cytoplasmic aggregates with increased orange fluorescence (Fig 12C). In contrast, the homogenous green cytoplasmic fluorescence in the ADJ group was completely replaced by large irregular orange aggregates irregularly distributed under the cell membrane, and randomly within the cytoplasm (Fig 12C). Further, the cells in the ADJ group more frequently assumed a unipolar morphology with the sub-membranous aggregates often concentrated away from an eccentric nucleus. Confocal imaging of these pulse-chase experiments demonstrate that treatment with ADJ alters antigen uptake and processing within DC2.4 cells, the intracellular localization of the antigen, and the cytomorphology of the DC2.4 cells.

To directly assess whether ADJ enhances the ability of DCs to activate and drive clonal expansion of naïve antigen-specific CD8 T cells, we pulsed DC2.4 cells with OVA mixed with PBS or ADJ for 30 minutes. Subsequently, antigen-pulsed DC2.4 cells were cultured with CFSE-labeled OT-I TCR transgenic CD8 T cells for 72 hours. Data in Fig 12D illustrate that DCs exposed to ADJ or OVA induced minimal proliferation of OT-I CD8 T cells. In striking contrast, DC2.4 cells exposed to OVA plus ADJ stimulated robust proliferation of OT-I CD8 T cells. Collectively data presented in Figs 11 and 12 indicate that ADJ treatment enhanced the ability of DC2.4 cells to uptake, process and present antigen to naïve CD8 T cells.

## Discussion

Here we report that ADJ, a Carbomer-lecithin-based adjuvant elicits potent mucosal and systemic cell-mediated immune responses to non-replicating antigens. We find that systemic CTL memory induced by parenteral immunization fails to confer protective immunity against influenza virus in the respiratory tract. Strikingly however, IN immunization with an experimental antigen or inactivated influenza A virus adjuvanted with ADJ induces CTL memory in the respiratory tract and confers robust protective immunity to influenza virus challenge. These findings provide key insights into the induction of CTL memory-dependent protective immunity by non-replicating antigens in the respiratory mucosa.

Our investigations into the kinetics of the CTL responses to prime-only and prime-boost protocols using the model antigen OVA revealed that booster vaccinations can dramatically enhance the magnitude of secondary effector responses. We also found that systemic and respiratory CTL memory resulting from SQ prime-boost vaccination is capable of mounting substantial recall responses in the spleen and lung, but these responses did not protect against challenge with influenza virus. Unlike antibodies that can rapidly and widely diffuse into areas of inflammation, memory CTLs are a heterogeneous population with divergent capacities for tissue trafficking and effector function and the initial programming of different CTL memory subsets affects the nature of their recall responses.[60] Why CTL recall responses generated by SQ vaccination with ADJ did not mediate enhanced viral control following respiratory viral challenge is unknown. It is possible that the nature of the systemic CTL memory is such that it could provide robust protection against systemic challenge. Alternatively, enhanced viral clearance occurs in ADJ/OVA vaccinated group at later time points after challenge (beyond day 6). We are designing additional experiments to explore these possibilities.

Because the phenotype of respiratory CTL memory is likely influenced by the nature of the initial challenge, we next investigated the viability of the intranasal route of vaccination with ADJ in mice. We find that SQ vaccination yielded substantial systemic CTL memory pools and limited respiratory CTL memory, while IN vaccination yields the reverse. Given the disparity in the anatomic distribution of the CTL memory resulting from SQ and IN vaccines, the magnitude of recall CTL responses in the lungs after influenza challenge was nearly identical with IN and SQ vaccination. However, further analysis show that improved viral control following IN vaccination and not SQ vaccination is associated with greater number of virus-specific CTLs in the airways. It remains to be determined whether airway CTLs are descendants of airway memory cells and/or memory cells found in the pulmonary vasculature, lung parenchyma or circulation. However, the association of greater number of memory T cells and secondary CTLs in airways with viral control in IN vaccinated mice is consistent with a report that airway memory CD8 T cells are necessary and sufficient for protection against influenza virus challenge.[61]

It has been previously reported that CTL-based mucosal immunity is greatly enhanced by the establishment of T_RM_, a unique subset of CTL memory that persists in the lungs.[62] T_RM_ is characterized by expression of the cell surface molecules CD103 and CD69, which facilitate the retention of memory CTLs within the lung parenchyma and airway epithelium.[62] Indeed, protection by IN vaccination not only correlated with a significantly larger pool of CTL memory cells within the lungs, but also with enhancement of T_RM_. Further, a greater proportion of the CTLs resulting from IN vaccination were capable of co-producing IFN-γ, TNF-α, and IL-2 suggesting a greater functional capacity as well. Significantly, only IN and not SQ vaccination elicited substantial number of memory CD8 T cells in the airways. Taken together, data presented in this manuscript strongly suggests that enhanced influenza virus control following IN vaccination with ADJ-OVA is linked to induction of T_RMs_ and airway memory CD8 T cells.

To address the concerns that responses to ADJ-OVA vaccines may not be representative of vaccines formulated with pathogen-associated antigens, we expanded our vaccine-challenge system to pathogen-associated antigens while minimizing confounding humoral immunity. For the antigen, we chose the BPL-inactivated A/PR8xTexas H3N2 strain of influenza virus, and A/PR/8/34 H1N1 strain for challenge. The PR8xTexas strain was derived from the PR8 strain, and is genetically identical to PR8 with the exception of HA and NA genes. We found that IN ADJ-PR8xTexas vaccines did indeed elicit robust CTL memory and recall responses in the lungs that were similar in magnitude to those previously generated by OVA-based vaccines. We observed that a minimum antigen dose is required to elicit these responses, and that higher doses did not necessarily elicit correspondingly larger CTL memory pools. The higher doses also provide substantial protection against heterosubtypic challenge, and the protection afforded by vaccine containing 3 μg HA (and other viral proteins) is equivalent to that of the 10 μg HA-containing vaccine preparation. In our analysis, we find that the 3 μg HA-containing vaccines generate slightly larger CTL responses than vaccines containing 10 μg HA, and that this correlates with increases in the number of virus-specific CD8 T cells producing IFN-γ and amount of IFN-γ produced by each cell, number and degranulation. Further, increased IFN-γ production correlated with increases in CD107a, which indirectly measures the capacity for degranulation and cytolytic function. Lastly, the 3 μg dose of HA yields the largest absolute number of CTLs expressing CD103 and CD69, suggesting that this dose is optimum for protection in this model, and that antigen dose may positively or negatively affect the generation of T_RM_. Why vaccination with greater amount of viral antigens reduces the number of memory CD8 T cells (especially T_RMs_) remains unknown. It is possible that higher levels of antigens might drive terminal differentiation of effectors and diminish the development of memory T cells in the respiratory tract. Because the antigen dosage of currently licensed influenza vaccines is based on HA content, we also formulated our vaccines based on HA concentration rather than the concentration of NP or other structural proteins. As NP protein may be twice as abundant as HA, it is likely that the actual concentration of antigen recognized by CTLs is substantially greater than suggested by HA concentration.[63] Therefore subsequent investigations would benefit from quantitation of specific antigens so that optimal doses can be determined. Further, our intent was to focus on the cell-mediated immune responses to the immunodominant NP epitopes in this model, and altered the HA and NA of the challenge virus to limit the contribution of potentially protective humoral responses targeting these proteins. We do recognize, however, that protection in this model is less constrained to CTL responses than the OVA model. ADJ can elicit potent antibody responses, and epitopes such as the conserved M1 and M2 proteins may be targets for protective humoral immunity.[64, 65] Nonetheless, we have clearly demonstrated that ADJ can elicit robust cell-mediated immune responses to non-replicating antigens.

The mechanisms by which adjuvants influence the generation of the CTL responses to non-replicating antigens are unclear.[14, 31] Some adjuvants appear to function primarily via signaling through innate pattern-recognition receptors (PRRs). This includes CpG DNA, which depends upon signaling via TLR9 receptors to activate antigen-presenting cells such as plasmacytoid dendritic cells to upregulate of antigen cross-presentation.[35, 52, 53] Others, such as the immune-stimulating complex adjuvant Iscomatrix^™^, may function independent of TLRs, yet but activate inflammasome signaling and alter intracellular localization of antigen in ways that facilitate antigen cross-presentation.[66, 67]

How ADJ elicits CTL responses is unknown. Recent studies on the immune-stimulating properties of ADJ found evidence that ADJ did not induce TLR or NLR signaling in vitro.[68] This does not preclude a role for these signaling pathways in the various interactions underlying the generation of CTL responses in vivo, therefore we repeated our SQ vaccine experiments in MyD88-deficient mice. Virtually all TLR signaling except TLR-3 is abolished in these mice, enabling us to evaluate the role of TLRs in CTL activation and primary responses. We find that primary CTL responses to ADJ were not affected by MyD88 deficiency. We did however note alterations in the SLEC:MPEC differentiation states of the primary CTLs in MyD88-deficient mice. Thus, ADJ might engage MyD88 signaling to promote differentiation of effector CD8 T cells.

APC activation affects both antigen-processing and migration to the secondary lymphoid tissues where naïve CTLs are activated. In the lymphoid tissues, the CTL activation depends on the amount, and strength, and duration of antigen signaling.[69] Therefore, we next looked at the effect of ADJ on APC activation, antigen uptake and processing, and migration in vivo. We find that SQ ADJ vaccination quickly increases the numbers of conventional DCs. A more extensive characterization of the immune cells in the airways (BAL) and lung following IN ADJ vaccination revealed much more profound changes in the composition of the inflammatory cell populations. Key alterations in the ADJ BAL were the recruitment of neutrophils, depletion of alveolar macrophages (aMΦ), and increased frequencies of exudative macrophages, inflammatory monocytes and inflammatory DCs. These findings suggest a strong pro-inflammatory environment in the BAL of ADJ-vaccinated mice. The aMΦ were not activated in either group, but the small population of CD103^+^ DCs in the ADJ group were strongly activated, expressing high levels of CD40, CD86, and MHC-II. In contrast to the BAL, the ADJ-treated lung displayed only modest alterations in the composition of innate immune cells. In preliminary experiments, transcription levels of inflammatory cytokines within the lung tissue revealed upregulation of IL-1, type I interferon, and TNF-α were strongly upregulated compared to PBS. Thus ADJ treatment induces a pro-inflammatory environment and significant alterations in the innate cell populations in the lung, with a dramatic shift from aMΦ to inflammatory DC’s, yet it is the small populations of CD103^+^ DCs that are highly activated. Since, migratory CD103^+^ DCs in lungs and intestines) are crucial for cross presentation in vivo[70, 71], it is possible that ADJ enhances the priming of CTLs in the lungs by activating this cell type.

To gain insights into ADJ’s mechanism of action, we investigated the effect of ADJ on the DC2.4 cell line, which is capable of direct and cross-presentation of antigen, and can fully activate CD4^+^ and CD8^+^ T cells.[57, 59] In an experiment where DC2.4 cells were continuously exposed to ADJ, DC2.4 cells displayed increased CD40 expression, a strong indicator of activation. In a pulse-chase model, the increase in CD40 expression persisted longer and to a greater degree in these cells, significantly longer than with LPS exposure. Interestingly, in the same experiment, ADJ markedly upregulated MHC-II expression, in the absence of antigen. While we did not find similarly dramatic alterations in global MHC-I expression on these cells, but ADJ did induce a small but detectable increase in the subset of MHC-I bearing SIINFEKL peptide. Further, in comparison to untreated cells, ADJ-treated DC2.4 cells potently stimulated the proliferation of naïve OT-I CD8 T cells in vitro. In sum, ADJ appears to augment antigen processing and presentation by DCs, at least in vitro.

Perhaps more impressively, in vitro studies with DQ-OVA found that antigen processing was upregulated by ADJ, with ADJ-treated cells exhibiting far greater primary and excimer fluorescence as evaluated by flow cytometry. Confocal imaging confirmed these findings, and demonstrated that the increased fluorescence was also associated with aberrant intracellular localization of the DQ-OVA. One of the putative key features of the Iscomatrix adjuvant is the capacity to rapidly translocate antigen to the cytosol.[67] It is possible that the diffuse green fluorescence spread throughout cells in the ADJ-treated group at 3 and 6 h post-treatment reflects a similar effect. The etiology of the irregular linear aggregates of DQ-OVA under the cell membrane in the ADJ group is unclear. Further investigations into the nature and behavior of ADJ-induced alterations in antigen uptake and processing are ongoing, with particular focus on co-localization studies and identification of the structures contributing to the excimer fluorescence observed with flow cytometry and confocal microscopy.

Still, the molecular mechanisms underlying the capacity for ADJ to elicit CMI are not clear. As the effects of ADJ appear to be MyD88-independent, and independent of other commonly recognized PRRs such as NLRs in vitro, additional mechanisms must be investigated. The components of ADJ themselves, polyacrylic acid polymers (carbomer) and soy lecithin may guide future studies. Polyacrylic acid (PAA), widely used in pharmaceuticals, has been previously shown to have potent antiviral effects in mice.[72, 73] This effect is tied to its chemical structure and its ability to elicit type I interferons when administered at a range of doses by multiple routes.[74]

The lecithin component of ADJ is largely composed of membrane phospholipids, primarily phosphatidylcholine and phosphatidylinositol.[75] In ADJ, lecithin is formulated as a nano-emulsion, and speculated to fuse with cell membranes similar to liposomes.[39] An additional possibility is that the inflammatory environment at the vaccination site results in the oxidation of these phospholipids. This could occur in the extracellular environment or within phagocytes, prior to being released during cell death. Oxidized membrane phospholipids are potent immunostimulatory molecules and signal through scavenger receptors CD36 and SR-B1 on macrophages and other innate cells.[76] Notably, CD36 signaling is TLR and integrin-independent, and involves signaling through fyn, p38 map kinase, JNK1 and JNK2.[77] In addition, CD36 signaling via SRC kinases leads to NFkB signaling and expression of IL-1 and TNF-α via NLRP3 inflammasome activation.[78] Thus the inflammatory responses to PAA and oxidized phospholipid-induced activation of CD36 are highly consistent with the findings in our study, particularly MyD88 independence, and rapid upregulation of type I interferons and TNF-α at the vaccination site. Additional mechanisms to consider are activation of the classical complement by interactions between phosphatidylcholine and C-reactive protein, and the abundant availability of phosphatidylcholines as a substrate for arachidonic acid metabolites.[79] Given the complex machinations of ADJ’s effect on innate immunity it is likely that one or more of these pathways are involved at different points in the generation of CTL memory.

In many of our studies, we compared the adjuvanticity of ADJ with Alum and CpG. While it is abundantly clear that ADJ is superior to Alum in eliciting primary, memory and recall CD8 T cell responses, ADJ and CpG induced comparable CD8 T cell responses to SQ vaccines. Regardless of the adjuvant used, CD8 T cell memory induced by SQ vaccination failed to provide protection against influenza virus in the respiratory tract. This reinforces the idea that vaccination by parenteral routes might not effectively program antibody independent CTL memory-dependent protective immunity in the respiratory tract. In this study, we did not compare the relative efficacies of ADJ and CpG in inducing protective immunity in the lungs following IN vaccination. Using a mouse model similar to ours, it has been shown that IN vaccination with CpG-OVA induced memory CTLs that reduced influenza virus replication in the lungs by ~2 logs, as compared to no adjuvant controls.[80] Thus, ADJ and CpG might program comparable levels of CTL-dependent protective immunity to influenza virus in the lungs. Future experiments will compare CTL-dependent protective immunity induced by ADJ and CpG.

Collectively, our studies present Adjuplex as a potent provocateur that manipulates key facets of the innate response to effectively generate cell-mediated immunity to non-replicating antigens. It does so by promoting the recruitment and activation of antigen-presenting cells to sites of vaccination, and induces local production of inflammatory cytokines leading to APC activation and likely further APC recruitment. Activated APCs in turn exhibit alterations in antigen uptake and processing, and enhanced trafficking of DCs to the DLN. The magnitude and character of the resulting CTL responses are strongly influenced by antigen and adjuvant dose, and route of vaccination. Indeed, vaccine route played a key role in the capacity for ADJ vaccines to generate protective mucosal immunity. The molecular mechanisms by which ADJ works remain to be discovered, however future investigations will likely yield vital insights into the conditions required for cell-mediated immune responses to non-replicating antigens.

## Materials and Methods

### Experimental animals

Six- to eight-week-old C57BL/6 (B6) mice were purchased from the National Cancer Institute (Bethesda, MD) or from restricted-access SPF mouse breeding colonies at the University of Wisconsin-Madison Biotron Laboratory. OT-I TCR Tg mice carrying Thy1.1 allele, OT-II TCR Tg mice carrying the Ly5.1 allele, and MyD88-deficient B6.129P2(SJL)-Myd88tm1.1Defr/J mice on the C57BL/6 background were provided by Dr. Bruce Klein (Department of Pediatrics, School of Medicine, University of Wisconsin-Madison, Madison, WI).[81, 82] All mice were housed in specific-pathogen-free conditions in the animal facilities at the University of Wisconsin-Madison (Madison, WI).

### Ethics statement

All experiments were performed in accordance with the protocol (Protocol number V1461) approved by the University of Wisconsin School of Veterinary Medicine Institutional Animal Care and Use Committee (IACUC). The animal committee mandates that institutions and individuals using animals for research, teaching, and/or testing much acknowledge and accept both legal and ethical responsibility for the animals under their care, as specified in the Animal Welfare Act (AWA) and associated Animal Welfare Regulations (AWRs) and Public Health Service (PHS) Policy.

### Bacteria, viruses, infection, virus inactivation, and titration

*Listeria monocytogenes* expressing chicken ovalbumin as a full-length protein (LM-OVA) was provided by Dr. Hao Shen (University of Pennsylvania School of Medicine, Philadelphia, PA). Mice were infected with 5x10^4^ CFU LM-OVA per mouse by tail-vein injection. Recombinant vaccinia virus expressing chicken ovalbumin as a full-length protein (VV-OVA) was provided by Dr. Jack Bennink (National Institutes of Health, Bethesda MD,).[83] Mice were infected with 5 × 10^5^ PFU VV-OVA per mouse by intraperitoneal injection. Influenza virus strain A/PR/8/34 H1N1 (PR8) and strain A/H1N1/PR/8/34 H1N1–OT-I (PR8-OVA), which expresses the SIINFEKL peptide of chicken ovalbumin, were a kind gift from Dr. Paul Thomas (St. Jude Children’ Research Hospital, Memphis, TN).[54] Influenza A strain PR8xTexas H3N2 (PR8-Tex), a reassortant virus composed of (A/PR8/H3N2 influenza virus containing HA [H3] and NA [N2] proteins from A/Texas/50/2012) was generated by reverse genetics in the Kawaoka Laboratory, as previously described[84]. The PR8-Tex virus was amplified by passage in eggs, and inactivated with 0.1% beta-propiolactone as previously described.[85] Inactivated virus was purified by sucrose gradient ultracentrifugation, and loss of infectivity was evaluated by inoculation into eggs. The viral genome was confirmed by sequencing, and the virus concentration was determined by Western blot for the hemagglutinin HA1 domain. For infection challenge studies, mice received a single intranasal inoculation of 500 PFU of PR8-OVA, and were humanely euthanized 6 days after infection. Lung tissues were frozen at −80°C in plain RPMI 1640 immediately after euthanasia for virus quantification. Tissues were rapidly thawed and homogenized in the RPMI media, and cleared supernatants were titrated on MDCK cells using standard methods.

### Adoptive transfer

For adoptive transfer experiments, spleens were harvested from OT-I TCR transgenic Thy1.1^+^ or OT-II TCR Tg Ly5.1^+^ mice. Spleens were mechanically processed into a single-cell suspension and erythrocytes were lysed by incubation with 0.9% NH_4_Cl for 1 minute. Then splenocytes containing 10^5^−10^6^ OT-I or OT-II TCR transgenic T cells were transferred to Thy1.2^+^ or Ly5.2+ mice, respectively, and mice were vaccinated 24 hours later.

### Vaccines and vaccination

Hen egg white ovalbumin grade V (OVA) was purchased from Sigma-Aldrich (St. Louis, MO). ODN 1826 CpG oligonucleotide adjuvant was purchased from InivivoGen (San Diego, CA), and was reconstituted in sterile phosphate-buffered saline (PBS). Adjuplex (endotoxin-free) was provided by Advanced BioAdjuvants, LLC (Omaha, NE). Imject^™^ Alum (ALM) was purchased from Thermo Fisher Scientific (Pierce, Rockford, IL). OVA antigen was prepared by dissolving crystallized OVA in sterile phosphate-buffered saline and passage through a 0.2 μm syringe filter. Ovalbumin or inactivated viruses were mixed with adjuvants by forceful pipetting and vortexing until homogenous, then aliquoted into 0.5 cc tuberculin syringes with 28g needles for intramuscular and subcutaneous injection, or individual 50 μl aliquots for intranasal inoculation via 200 μl pipette.

For intramuscular vaccines, 25 μl of vaccine was injected bilaterally into the tibialis muscles as previously described.[86, 87] For subcutaneous vaccines, the tail base was cleaned with 70% ethanol and 50 μl of the vaccine was administered subcutaneously. For intranasal vaccinations, mice were briefly anesthetized with 3% isoflurane in oxygen and the vaccine was slowly inoculated into the nares.

### DC2.4 activation and antigen-processing assays

The immortalized DC2.4 dendritic cell-like line was a gift of Dr. Kenneth Rock (Department of Pathology, University of Massachusetts Medical School, Worcester, MA) DC2.4 cells were maintained in DMEM high glucose (4500 mg/L, Life Technologies) supplemented with 10% fetal bovine serum, 100 U/ml penicillin G, 100 g/ml streptomycin sulfate, and 50 μM 2-ME.

For in vitro cell assays, FITC-conjugated chicken ovalbumin (FITC-OVA) and DQ-OVA (Molecular Probes) were purchased from Life Technologies, Inc. and reconstituted in DC2.4 growth media. 1 x 10^6^ DC 2.4 cells were plated per well in a 96-well plate and grown overnight at 37C 5% CO_2_. The media was aspirated and replaced growth media only, or growth media containing FITC-OVA (200μg/mL), DQ-OVA (200 ug/mL), FITC-OVA + LPS (5 μg/mL) with and without Adjuplex 1% V/V. For pulse-chase experiments, the treatment media was washed off after 30 minutes and completely replaced with growth media every 24 hours. For other experiments, DC2.4 cells were incubated in the treatment media for the indicated time. After treatment, cells were harvested, washed 3 times, stained for viability with an amine-reactive dye as indicated below, incubated with Fc-Block (BD Biosciences, San Diego, CA) in PBS at a 1:200 dilution for 15 minutes, and stained with fluorochrome-conjugated antibodies as below.

### Flow cytometry

Single-cell suspensions of mononuclear cells from lymph nodes, spleen, lung, and bronchoalveolar lavage were prepared using standard techniques. Briefly, prior to antibody staining, some cells were stained for viability with either Fixable Viability Dye eFluor^®^ 506 or eFluor^®^ 780 (eBiosciences, San Diego, CA), or Ghost Dye^™^ Red 780 (Tonbo Biosciences, San Diego, CA) according to manufacturer’s instructions. Fluorochrome-labeled antibodies against the cell-surface antigens Thy1.1, Thy1.2, Ly5.1 (CD45.1), Ly5.2 (CD45.2), CD4, CD8a, CD8b, CD44, CD62L, KLRG-1, CD127, CD103, CD69, CXCR3, CD11b CD11c, CD40, CD80, CD86, Siglec-F, F4/80, Gr-1, Ly6C, Ly6G, and intracellular antigens IFN-γ, TNF-α, IL-2, CD107a, T-bet, and Eomes were purchased from BD Biosciences (San Jose, CA), Biolegend (San Diego, CA), eBioscience (San Diego, CA), or Tonbo Biosciences. The antibody recognizing the H2-K^b^:SIINFEKL complex was purchased from eBiosciences.[88] The anti-granzyme B antibody was purchased from Invitrogen (Grand Island, NY). Fluorochrome-conjugated H2-K^b^ tetramers bearing the ovalbumin peptide SIINFEKL (OT-I), and H2-D^b^ tetramers bearing influenza nucleoprotein peptide ASNENMETM (NP366) and acidic polymerase peptide SSLENFRAYV (PA224) were obtained from the NIH Tetramer Core Facility (Emory University, Atlanta, GA). Cells were incubated with tetramer for 60 minutes on ice in the dark, and with antibodies for 30 minutes on ice in the dark. Intravascular staining for vascular CD8 T cells in the lungs was performed as previously described. [89] Briefly, 5 minutes prior to euthanasia, mice were infused with fluorochrome-labeled anti-CD8β antibodies. Cells from lungs were stained with anti-CD8α and other surface markers. Cells positive for both CD8α and CD8β were considered as vascular CD8 T cells.

### Intracellular cytokine stimulation

For intracellular cytokine staining, 1x10^6^ cells per well were plated on flat-bottom tissue-culture-treated 96-well plates. Cells were stimulated for 5 hours at 37C in the presence of human recombinant IL-2 (10 U/well), and brefeldin A (1 μl/ml, GolgiPlug, BD Biosciences), with one of the following peptides: SIINFEKL, NP366, PA224 (thinkpeptides^®^, ProImmune Ltd. Oxford, UK) at 0.1ug/ml, or without peptide. After stimulation, cells were stained for surface markers, and then processed with Cytofix/Cytoperm kit (BD Biosciences, Franklin Lakes, NJ). Permeabilized cells were transferred to FACS buffer for acquisition, while surface-stained cells were fixed with 2% paraformaldehyde in PBS for 20 minutes, then transferred to FACS buffer. All samples were acquired on FACSCalibur, LSR II, or LSRFortessa (BD Biosciences) analytical flow cytometers. Data were analyzed with FlowJo software (TreeStar, Ashland, OR).

### Confocal microscopy

For confocal microscopy, 1x10^4^ DC2.4 cells were plated in growth medium on 12mm diameter #1.5 coverslip glasses (Warner Instruments, Hamden, CT) in 24-well tissue culture plates (Corning Costar, Sigma Aldrich). Cells were grown overnight, and treated as described for activation and antigen-processing assays. After treatment, the coverslip glass was washed three times with Dulbecco’s PBS containing Ca++/Mg++ (DPBS, Life Technologies), fixed in 1% PFA in DPBS for 10 minutes, washed 3 times with DPBS, permeabilized with 0.1% saponin at room temperature, and incubated with DAPI (Life Technologies) at 1ug/ml for 5 minutes at room temperature. Cover glass was mounted in Vectashield (Vector Laboratories) and mounted on Permafrost microscope slides (Thermo Scientific). Cells were imaged on a Leica SP8 confocal laser-scanning microscope within 48 hours of mounting.

### Histopathology

Following euthanasia, tissues surrounding IM and SQ injection sites were collected en-bloc and fixed in 10% neutral phosphate-buffered formalin (NBF, Sigma-Aldrich). Lungs were perfused in situ by intratracheal administration of 750 μl NBF, then the trachea was ligated, and the lungs and heart were removed en bloc and immersed in NBF. Preserved tissues were paraffin embedded, replicates of 5-μm-thick sections were prepared for each tissue, and sections were stained with standard hematoxylin and eosin. Tissue sections were evaluated histologically by a board-certified veterinary anatomic pathologist (DJG), and photomicrographs were created with an Olympus BX41 microscope, DP71 camera system, and cellSens software (Olympus, Tokyo, Japan).

### Statistical analyses

Data statistics were calculated with Prism version 6.0g for Mac OS X (GraphPad Software, La Jolla California USA, www.graphpad.com). Student’s two-tailed *t-*test, and one-way ANOVA analyses were used to calculate the statistical significance of differences between groups, and significance was defined at *p* < 0.05.

## Supporting Information

S1 Fig ADJ Does Not Cause Pathological Alterations in the Lungs. Lung histology of mice following intranasal administration of 50 μL of PBS only (A, C, E) or with 10% Adjuplex (B, D, F). At 24 hours following administration there are no significant histological changes with PBS alone (A), or with a Adjuplex (B). Seven days following administration, there were no significant changes with PBS alone (C), however perivascular cuffs of lymphocytes and a mild increase in the number of alveolar macrophages are evident following Adjuplex administration (D). At 63 days following administration, there were no significant histological changes in with PBS alone (E), or with Adjuplex (F).
(TIF)

S2 Fig Lung Secondary CD8 T Cell Responses to Prime-Boost SQ or IN Vaccination. C57BL/6 mice were immunized intranasally with 10 μg of OVA in 50 μl PBS with 5% ADJ (SQ) or 10% ADJ (IN) twice at 3 week intervals. At 21 days post-boost, 5 mice/group were infected by IN administration of PR8-OT-I, and 6 days later we quantified secondary CD8 T-cell responses in the lungs. Data is representative of two independent experiments.
(TIF)

S3 Fig Recall Responses in Lung Following SQ and IN Vaccination with ADJ-OVA. Mice were vaccinated with ADJ-OVA by the SQ or IN route. At 21 days after vaccination, mice were challenged by IN administration of 500 PFU of recombinant influenza A/PR/8/34-OT-I H1N1 expressing the OVA SIINFEKL peptide. 6 days after challenge, 3–5 mice/group were sacrificed and BAL and lungs were collected to quantify SIINFEKL-specific CTLs using MHC I tetramers. Graph shows the total number of SIINFEKL-specific CD8 T cells in lungs and BAL.
(TIF)

S4 Fig Effect of Intranasal Adjuplex Administration on the Cell Populations in Bronchoalveoloar Lavage Fluid and Lung Tissue. Mice were vaccinated by IN inoculation of 50μl PBS with and without 10% ADJ, and bronco-alveolar lavage (BAL) fluid and lungs were collected 24 hours later. Data shows cell recovery at 24h after vaccination. Data is representative of two independent experiments.
(TIF)

S5 Fig Gating Paradigm for Identifying Inflammatory Cell Subsets in the Lungs by Flow Cytometry. Dichotomous branching indicates sequential steps for identification of each subset of cells.
(TIF)
