# Supplementary figures and images for: Effective Respiratory CD8 T-Cell Immunity to Influenza Virus Induced by Intranasal Carbomer-Lecithin-Adjuvanted Non-replicating Vaccines
Source: PLoS Pathog. 2016 Dec 20;12(12):e1006064. doi: 10.1371/journal.ppat.1006064 (PMC5173246; doi:10.1371/journal.ppat.1006064)

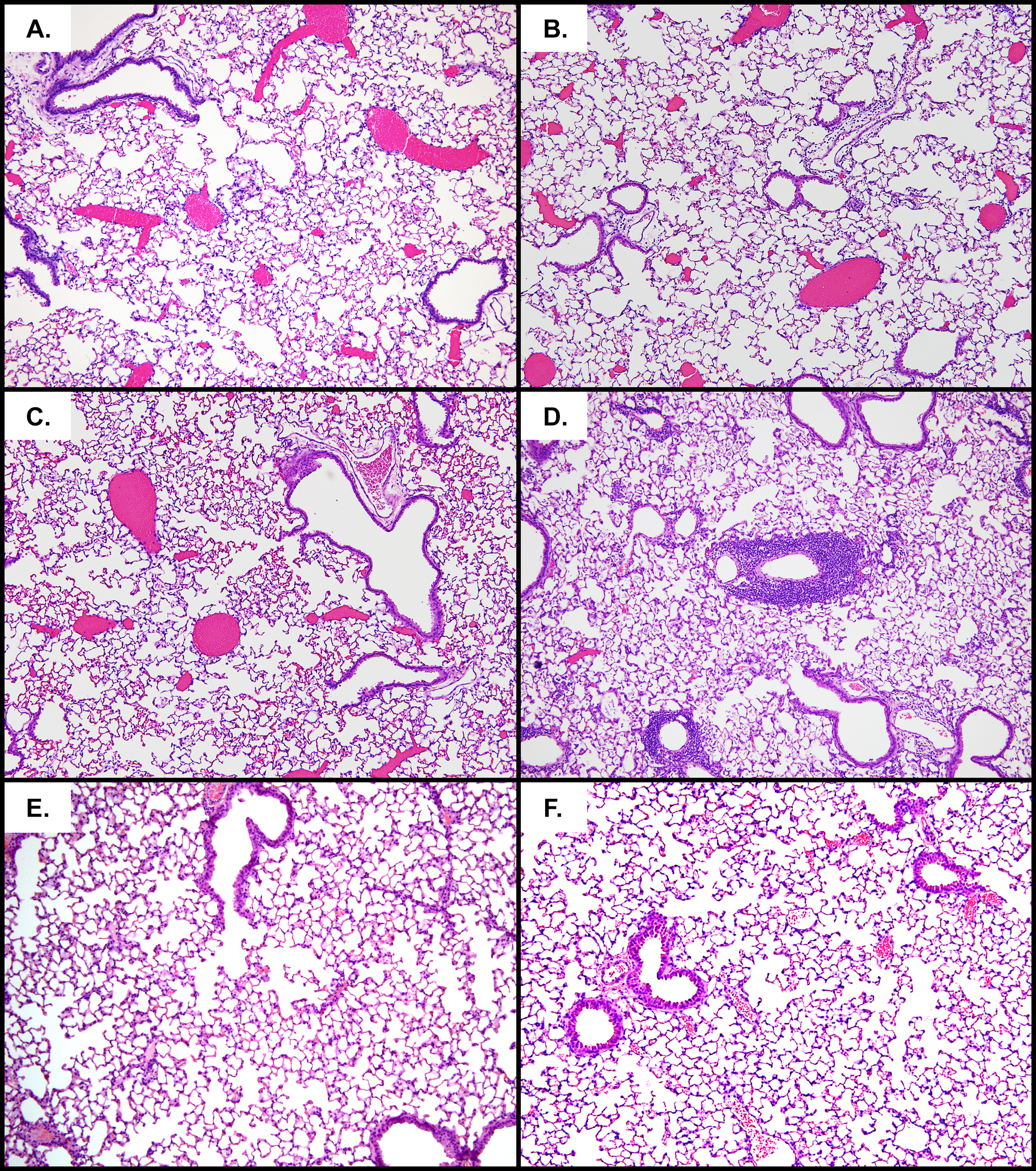

Supplement: S1 Fig — Lung histology of mice following intranasal administration of 50 μL of PBS only (A, C, E) or with 10% Adjuplex (B, D, F). At 24 hours following administration there are no significant histological changes with PBS alone (A), or with a Adjuplex (B). Seven days following administration, there were no significant changes with PBS alone (C), however perivascular cuffs of lymphocytes and a mild increase in the number of alveolar macrophages are evident following Adjuplex administration (D). At 63 days following administration, there were no significant histological changes in with PBS alone (E), or with Adjuplex (F). (TIF) [file ppat.1006064.s001.tif]

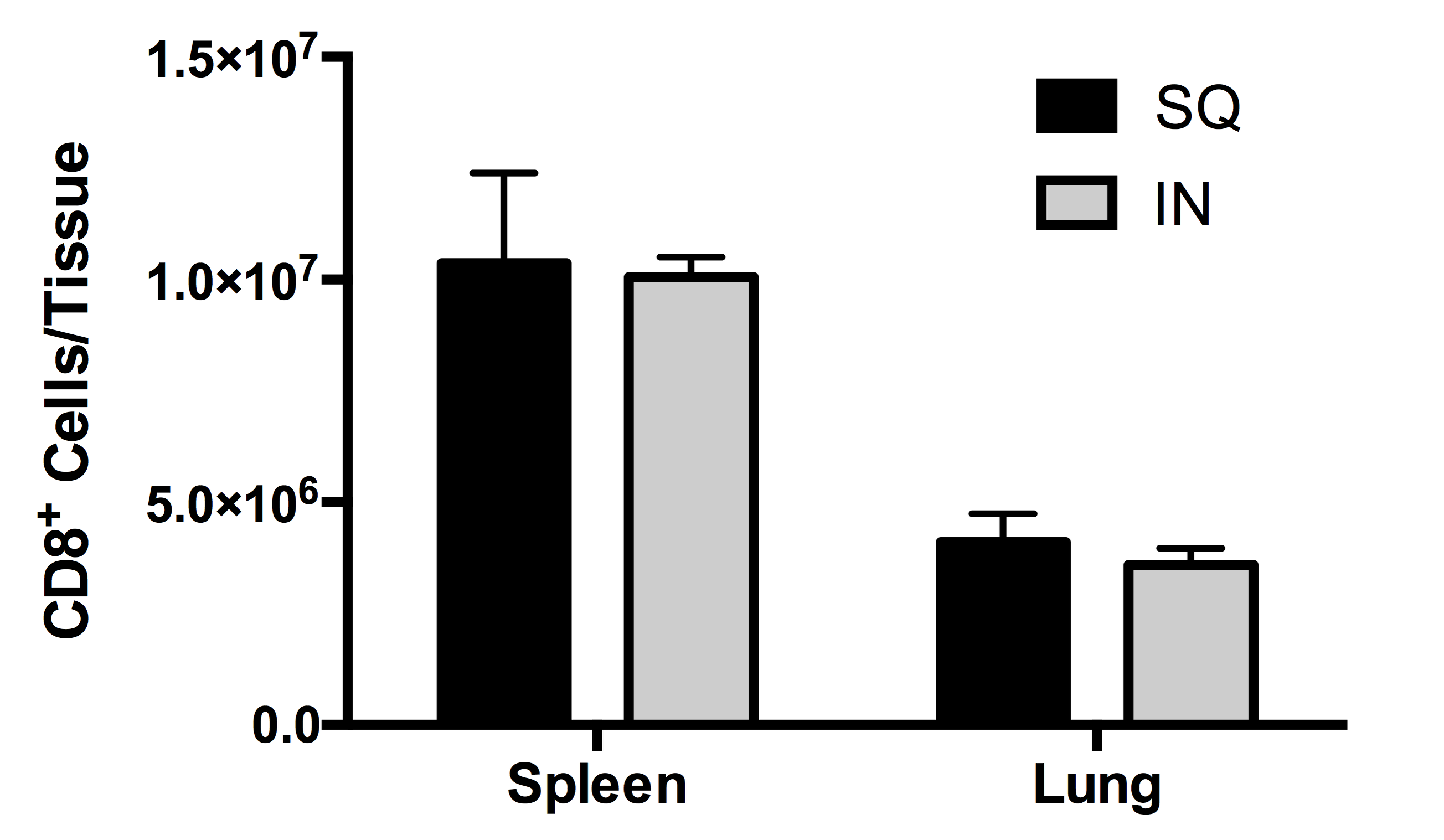

Supplement: S2 Fig — C57BL/6 mice were immunized intranasally with 10 μg of OVA in 50 μl PBS with 5% ADJ (SQ) or 10% ADJ (IN) twice at 3 week intervals. At 21 days post-boost, 5 mice/group were infected by IN administration of PR8-OT-I, and 6 days later we quantified secondary CD8 T-cell responses in the lungs. Data is representative of two independent experiments. (TIF) [file ppat.1006064.s002.tif]

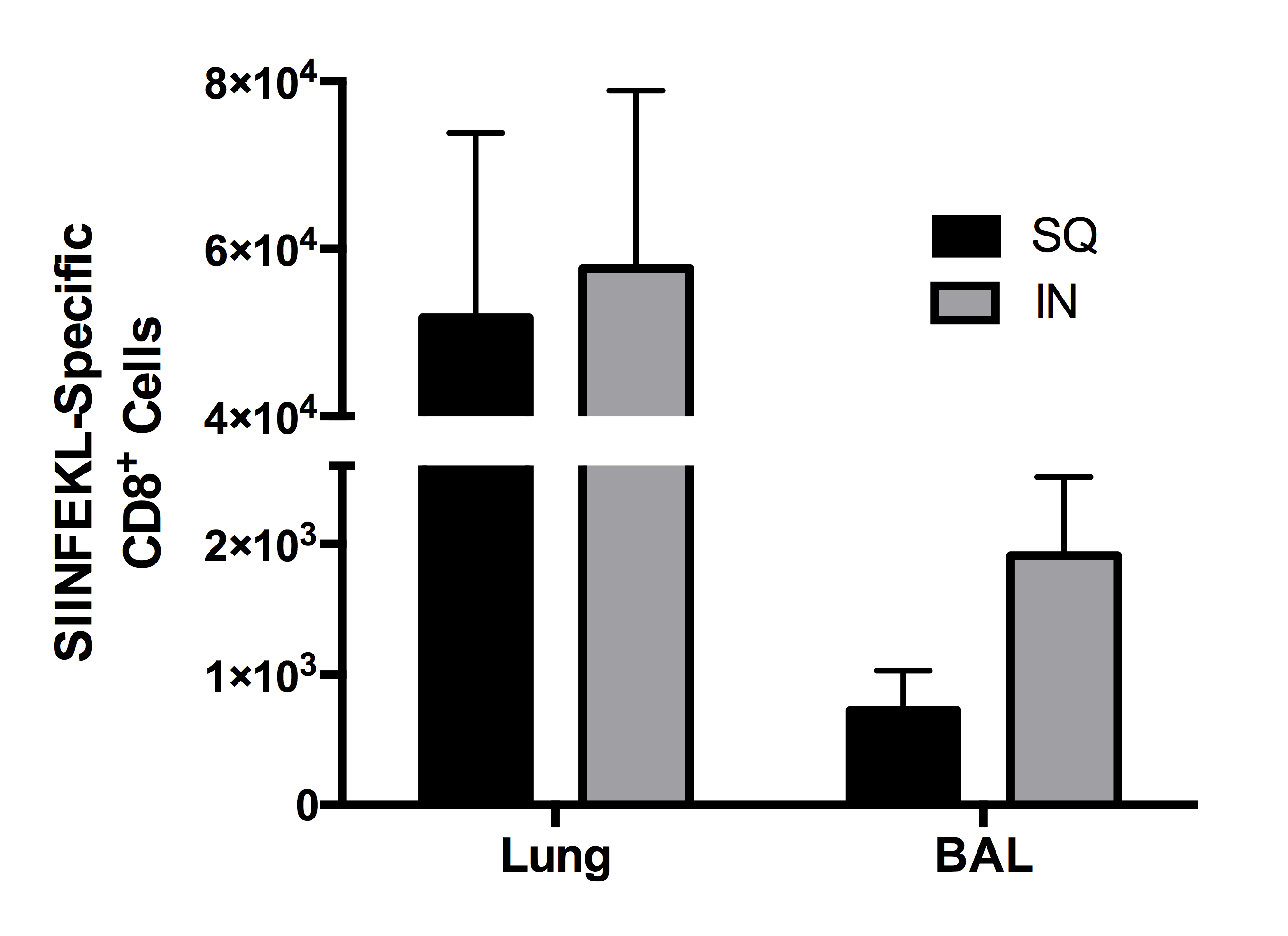

Supplement: S3 Fig — Mice were vaccinated with ADJ-OVA by the SQ or IN route. At 21 days after vaccination, mice were challenged by IN administration of 500 PFU of recombinant influenza A/PR/8/34-OT-I H1N1 expressing the OVA SIINFEKL peptide. 6 days after challenge, 3–5 mice/group were sacrificed and BAL and lungs were collected to quantify SIINFEKL-specific CTLs using MHC I tetramers. Graph shows the total number of SIINFEKL-specific CD8 T cells in lungs and BAL. (TIF) [file ppat.1006064.s003.tif]

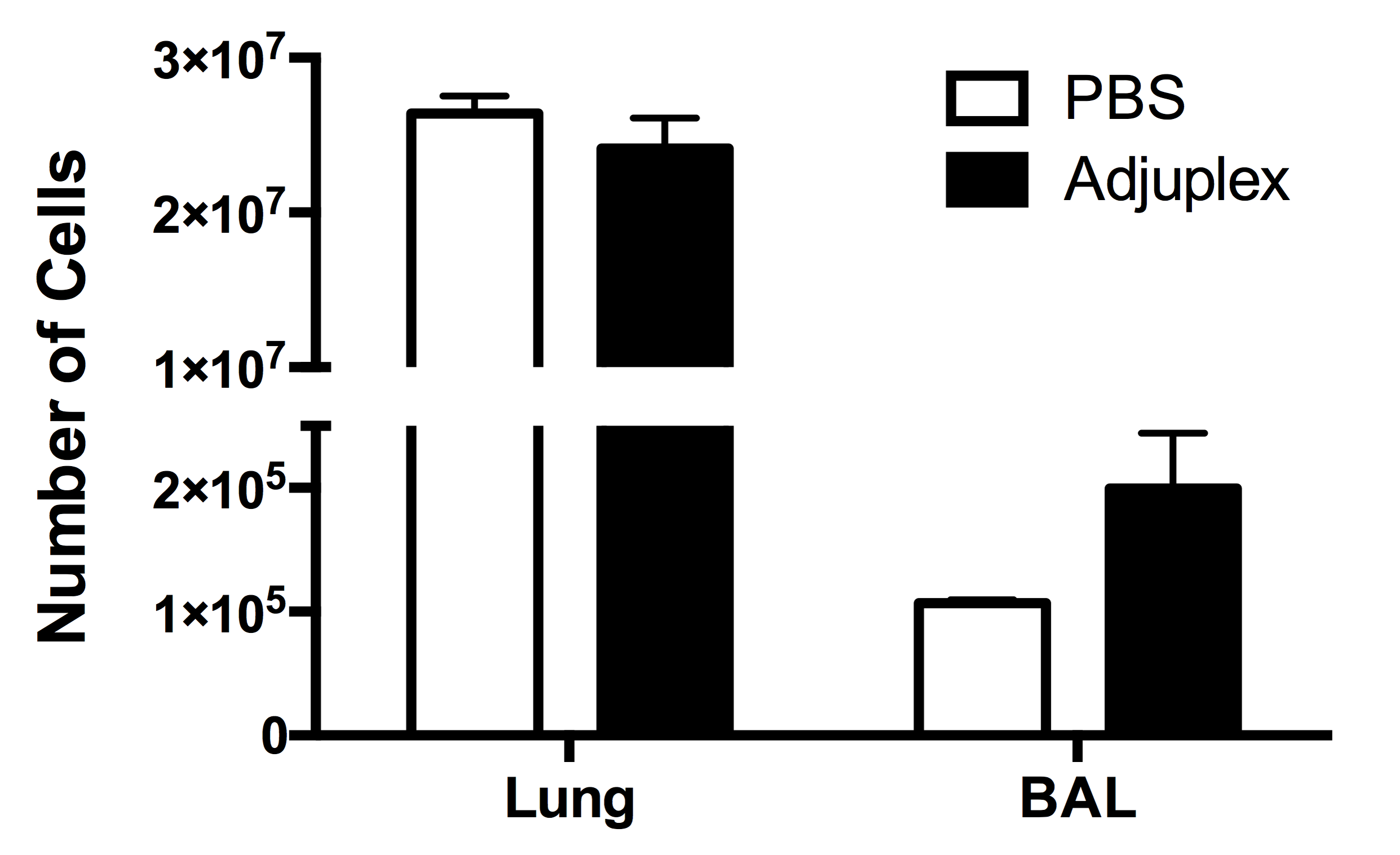

Supplement: S4 Fig — Mice were vaccinated by IN inoculation of 50μl PBS with and without 10% ADJ, and bronco-alveolar lavage (BAL) fluid and lungs were collected 24 hours later. Data shows cell recovery at 24h after vaccination. Data is representative of two independent experiments. (TIF) [file ppat.1006064.s004.tif]

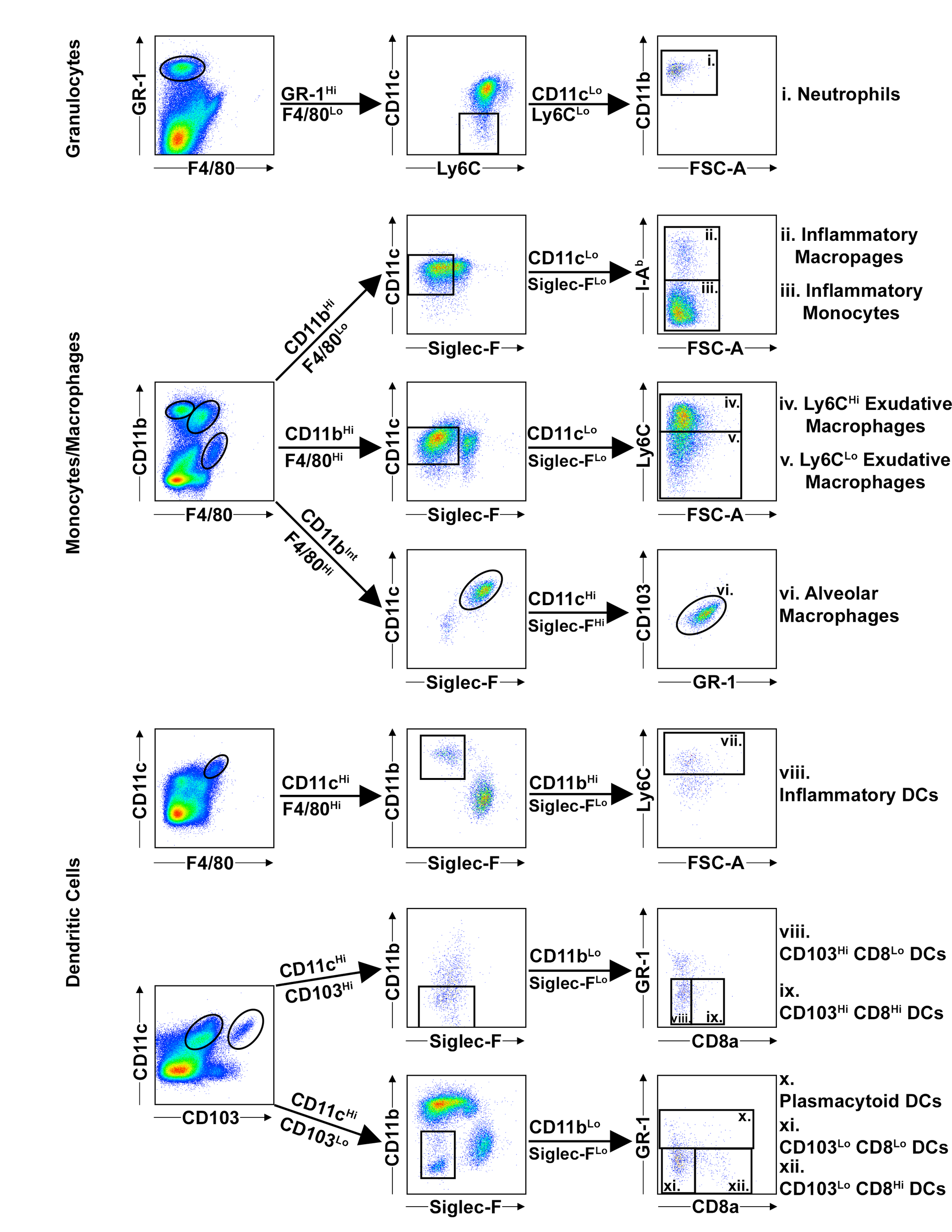

Supplement: S5 Fig — Dichotomous branching indicates sequential steps for identification of each subset of cells. (TIF) [file ppat.1006064.s005.tif]
